# A Shear Thickening Colloidal Suspension Functioning via Progressive Impact Jamming with Persistent Lubrication Layer

**DOI:** 10.1002/advs.202519754

**Published:** 2026-01-20

**Authors:** Yiran Wu, Yifeng Yu, Yiqiu Zhao, Qin Xu, T. X. Yu, Xin Zhang, Jinglei Yang

**Affiliations:** ^1^ Department of Mechanics and Aerospace Engineering Southern University of Science and Technology Shenzhen China; ^2^ Department of Mechanical and Aerospace Engineering The Hong Kong University of Science and Technology Clearwater Bay Hong Kong SAR China; ^3^ HKUST Shenzhen–Hong Kong Collaborative Innovation Research Institute Futian Shenzhen China; ^4^ Department of Physics The Hong Kong University of Science and Technology Clearwater Bay Hong Kong SAR China

**Keywords:** ionic liquid‐based shear thickening fluid, lubrication layer, modified added mass model, progressive jamming

## Abstract

From strong shear thickening (ST) to jamming, conventional shear thickening fluid (STF) systems have been thoroughly explored in the past few decades. A picture from hydrodynamics to lubrication breakdown and interparticle friction has been unveiled and verified in various studies for conventional STF systems. This work presents a well‐studied ionic liquid‐based shear thickening fluid (ILSTF) in an unexplored field of jamming dynamics, which demonstrates a lubricated nature not only in strong shear thickening, as characterized by the rheology test, but also in the jamming and even solidification process triggered by dynamic normal impact. A well‐fitted Modified Added Mass Model is built for jamming with the solid boundary, based on the classical Impact‐Activated Normal Solidification (IANS) framework. Impact jamming front propagates with a few nanometers’ thickness solvation layer lubricating the silica nanoparticles, before it decreases due to the squeezing induced by the gradual compression of increasing stress level on the jammed zone. This can be classified as a progressive jamming realm.

## Graphical abstract

An ionic liquid‐based shear‐thickening fluid (ILSTF) exhibits lubricated dynamics from strong shear thickening to impact‐triggered jamming. A persistent nanometric solvation layer around silica governs both the shear thickening saturation in rheology and jamming front propagation under dynamic impact compression. A Modified Added Mass Model within the IANS framework quantifies progressive jamming and boundary effects, explaining transient solidification and enhanced impact energy absorption.

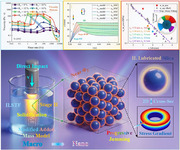

## 1 Introduction

Shear‐thickening dispersions exhibiting self‐adaptive impact absorption properties have attracted significant research interest for decades [1, 2, 3, 4]. The relationships and transitions between strong continuous and discontinuous shear thickening (CST& DST) [5, 6, 7, 8], to shear jamming (SJ) [9, 10, 11, 12, 13, 14], and even the isotropic jammed state [15, 16, 17], have drawn great interest, especially over the past two decades. Water‐cornstarch [18, 19, 20, 21, 22] and (poly)ethylene glycol (EG/PEG)‐based systems [23, 24, 25, 26, 27, 28, 29] are well‐explored. However, the jamming transition and dynamic performance of ionic liquid (IL)‐based dispersions, as another typical category of STF [30, 31], remain rarely explored. ILs offer extreme environment tolerance and multifunctionality, useful in various applications such as functional solvents, catalysts, lubricants, and electrolytes [32, 33, 34]. Thus, ILSTF is anticipated to be a smart, multifunctional material system, extending beyond impact energy absorption [35].

However, the physical nature of shear thickening to jamming behaviors in ILSTFs still requires further exploration, particularly in dynamic transient processes such as impact jamming, despite several preliminary studies [31, 36]. Starting with the static condition, the stabilization mechanisms of the dispersion of nanoparticles in ionic liquids are amply explored. For example, hydrophilic nano‐silica is well dispersed in [BMIm][BF_4_], verified with solvation layers providing repulsive forces [37]. Similarly, hydrophilic nano‐silica in hydroxyl PEG/ polypropylene glycol (PPG) demonstrates stable dispersion and shear thickening, whereas methyl‐terminated fluid molecules exhibit flocculation and shear thinning [38]. Interestingly, there is hydrogen bonding in both dispersions between the surface silanol group of the nano‐silica and the molecule of the liquid media [39]. Hence, we can reasonably deduce the hypothesis that the hydrogen bonding contributes to the strong shear thickening and jamming transition in ILSTF. The truth seems to be on the verge of being revealed, especially after recent findings that hydrogen bonding is capable of eliciting shear jamming via affected interparticle friction [40, 41]. As the lubrication to frictional contact transition is regarded as the DST and SJ origin [42, 43, 44], the above hypothesis is further bolstered.

But still, several critical questions remain unanswered, requiring further exploration. First, whether the strongest shear thickening of ILSTF belongs to DST, especially with the occurrence of the inspected lubrication layer. Besides, what would happen if an increased particle fraction enabled the interfered lubricated spheres during shear thickening? Second, why does the permittable particle fraction vary when crossing a wide range of solid particle dimensions? Or could the lubricated nature explain the size‐dependent DST/jammed particle fraction? Third, what role does the lubrication layer play during a much more severe transient jamming process, from shear jamming to solidification triggered by direct impact? Would the jamming front propagation be dominated by bare particles or lubricated ones?

In this study, the aforementioned questions are addressed, far beyond prior explorations of the ILSTF system [31, 36, 39]. Three aspects of side proofs are unified in a common physical picture: a lubrication layer of [BMIm][BF_4_] around nano‐silica of a thickness around 4–5 nm, effectively repulsed with near‐frictional contact during shear thickening and jamming, then squeezed thinner during solidification. This not only accounts for the low capacity of particle fraction in the dispersion toward forming solidity but also elicits the shear thickening saturation effect upon critical particle content. Furthermore, the imaginary impact‐activated jamming front propagation is governed as well. Building on this mechanistic picture, the translation to engineering impact‐protection applications based on this promising multifunctional system is now within reach.

## 2 Results and Discussion

### 2.1 Rheology Characterization

**FIGURE 1:**
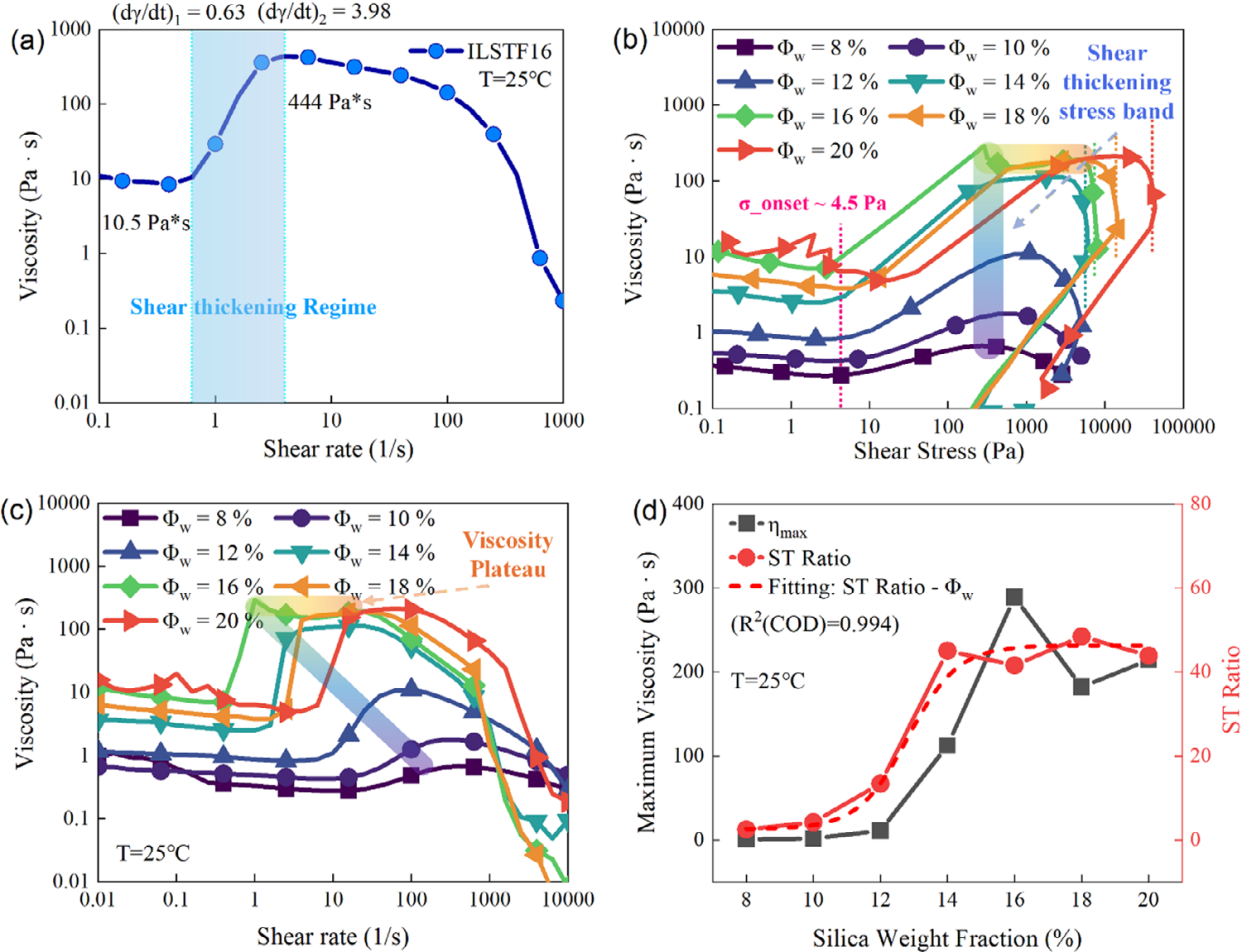
The rheology performance of the ILSTF at various nano‐silica mass fractions. (a) The rheology curve of the optimized ILSTF (Silica weight fraction (Φ_*w*_) equals to 16 wt.%) of the current composition under room temperature, with the viscosity right before and after the shear thickening regime marked out. (b) Viscosity—shear stress curves of various silica mass fractions under room temperature, with common shear thickening onset stress, maximum shear stresses (of near vertical turn‐down curves), shear‐thickened stress band, and the viscosity plateau marked out, as well as the shear thickening strength β of each curve. (c) Viscosity‐ shear rate curves of various silica mass fractions marked with two bands. 30 raw data points of each curve in b & c marked by typical symbols only. (d) The silica fraction dependence of the maximum viscosity & the shear thickening ratio are plotted simultaneously, with a dose‐response curve fit.

As shown in Figure 1a, the rheology curve of the optimized weight fraction ILSTF denotes a shear thickening ratio (defined by the maximum viscosity over onset viscosity of shear thickening) over 40 and continuous shear stress increase until two magnitudes higher shear rate above the onset shear rate. Then the thickening is followed by the second shear‐thinning stage, denoting the structural breakdown of the thickened system under further shear.

Upon closer inspection of the viscosity‐stress plot, a shear stress‐dominating framework is revealed in ILSTF rheology. As demonstrated in Figure 1b, a common onset stress of shear thickening of various silica weight fractions is witnessed, near 4.5 Pa at room temperature, even matching the DST lower bound in the shear stress‐particle fraction diagram [10]. Whereas when approaching the maximum viscosity, a plateau occurs (beyond 14 wt.% silica fraction) for each curve. Beyond this plateau, the viscosity drops precipitously once the shear stress approaches the maximum allowable value (τ_*ma*_) for the given silica fraction. The stress level is positively related to the silica fraction, of a magnitude from several to dozens of kPa, corresponding to the previous STF rheology results. Hydrodynamic interaction is explicable for the Pa to kPa level particle transient aggregation, leading to an abrupt viscosity increase. Then the viscosity saturation illuminates the embodied realities of a novel shear jamming paradigm of silica fraction from 16 wt.% onward. Under pure shear loading, the system approaches jamming at reduced interparticle distances due to increased concentration. Beyond this point, the rise in viscosity gives way to a regime dominated by shear‐induced jamming. In this state, clusters of nanoparticles reorganize into force‐bearing chains, which persist until the structure collapses upon exceeding a critical shear stress threshold.

Moreover, the system is observed to have a strong, but still continuous shear thickening (β < 1, in which the shear thickening strength β = log (Δη)/log (Δσ) as defined in a previous study [45]. The detailed maximum allowable shear stress and shear thickening strength values of dispersions exhibiting strong shear thickening are listed in Table 1 below, corresponding to those in Figure 1b. As frictional interaction is necessary in discontinuous shear thickening behavior, the IL‐lubricated spheres of strong interactions still permit more room compared to the pure frictional contact case. Thus, the strong continuous shear thickening of ILSTF aligns with its supposed physical framework. This is further bolstered by the additional rheological analysis in Section S1 (Section S1) where the system is hydrodynamically dominated with slight frictional particle interactions.

**TABLE 1:** Particle mass fraction—Dependent indices: Shear thickening strength (β) and maximum allowable shear stress (τ_*ma*_).

Amounts	Values				
Φ_*w*_ ^a^ ^)^	12 %	14 %	16 %	18 %	20 %
β	0.60	0.87	0.80	0.88	0.78
τ_*ma*_ (kPa) ^b^ ^)^	/	5.6	7.4	13.9	40.1

^a)^ Particle mass fraction in ILSTF dispersions (exhibiting strong ST) in rheology test.

^b)^ Maximum allowable shear stress (the plateau marked by vertical dotted lines in Figure 1b).

Returning to the rheology performance characterized by a wide range of silica fractions, the shear thickening of ILSTF originates at a much lower limit of 8 wt.%. The increased nano‐silica mass fraction of ILSTF is observed in Figure 1c,d, with the enhanced shear thickening effect in the range of 8 wt.% ~ 14 wt.%. In Figure 1c, beyond the latter concentration, a sharp transition is witnessed for each viscosity, together with a plateau before another shear thinning of a much higher shear rate. The transition point near the maximum viscosity then shifts right beyond 16 wt.%, a different mode compared to the conventional up‐left shifting, as explained to be a paradigm shift. This mode transition marks a thickening saturation phase characterized by a constant viscosity plateau and a higher shear‐thickened onset stress at its start. A lubrication layer thickness is estimated to be *t*_*lub*1_ = 4.60 nm (detailed derivation in Section S2, when considering this 16 wt.% equivalent to the previous particle volume fraction Φ ∼ 0.52 in the STF diagram of a corresponding stress level (290 Pa) entering shear jamming [10]. Based on *t*_*lub*1_, a Mooney model of suspension relative viscosity is well‐fitted of < 16 wt.% fraction below ST onset, providing additional validation (see Section S3, including Table S1).

### 2.2 Stable Dispersion Analysis

**FIGURE 2:**
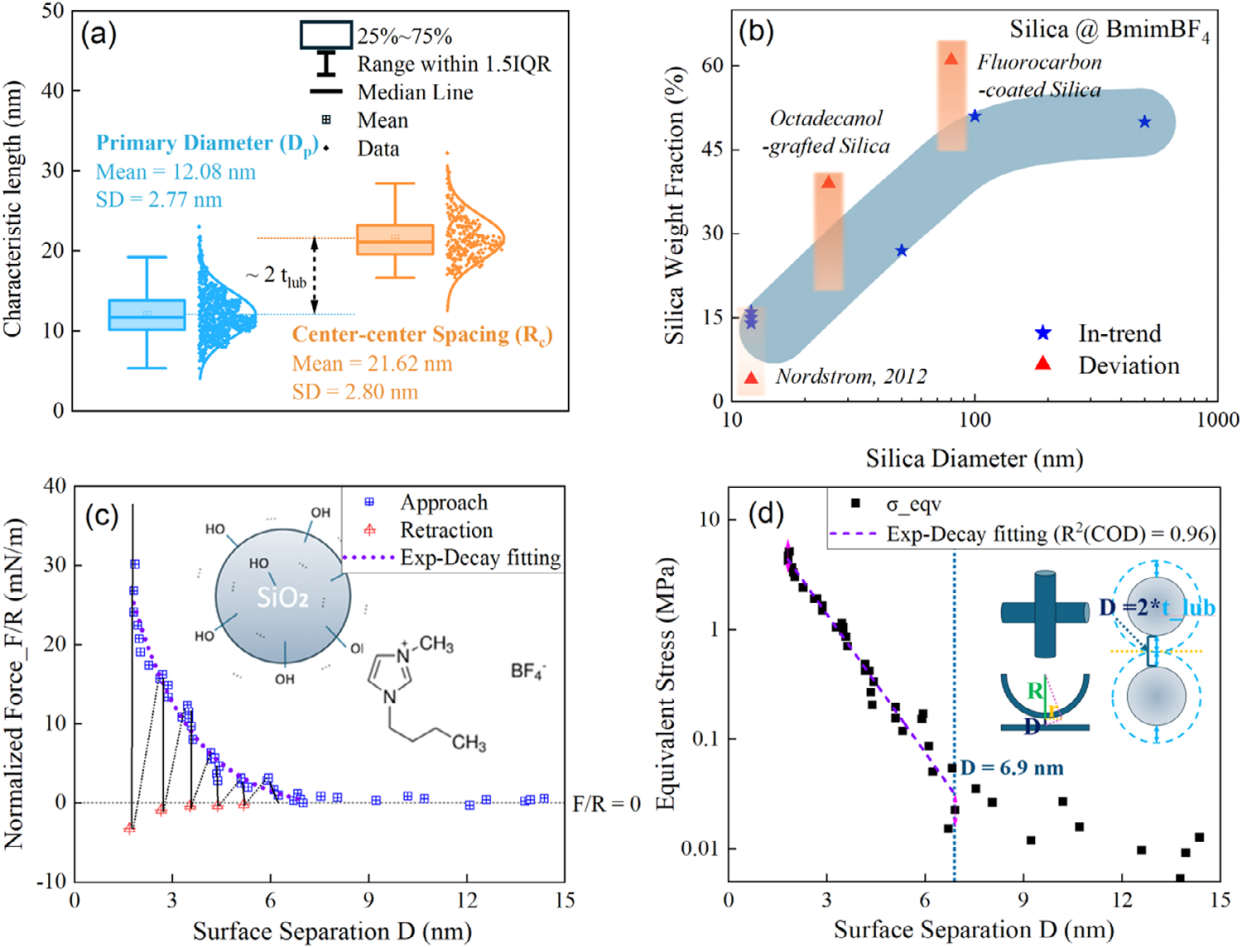
Particle diameter, size dependence fraction, and particle interaction of ILSTF in static conditions. (a) Violin–box plots of silica primary diameter (TEM, raw powder) and center–center spacing in IL (cryo‐TEM). Box = IQR (25%–75%), whiskers = 1.5×IQR, line = median, square = mean). (b) Maximum reported silica mass fractions in [BMIm][BF_4_] dispersions from literature (see Section S6). (c) SFA‐measured curvature‐scaled normal forces vs. surface separation between two vertical columns in [BMIm][BF_4_], as referred to with a schematic of nano‐silica and [BMIm][BF_4_] structure [48]. (d) Derived equivalent normal stress (log‐scale)—separation curve with ‘linear’ fit (appearing linear in log scale‐Y, exponential decay in real) in effective range (*D* = 6.9 nm). Upright inset: SFA columns (left, the case in referred result) vs. approaching spheres (right, the case in the current study) schematic. Adapted with permission from Ref. [48]. [2025] [Royal Society of Chemistry]. All rights reserved.

TEM photos (sample photo in Section S4 of Aerosil‐200 nano‐silica are utilized for accurate calibration of its size distribution of over 200 particles. We obtain an average diameter of 12.08 nm with a standard deviation of 2.77 nm, as shown in Figure 2a. Other basic physical constants are also listed in Section S5 (Table S2). Here we summarize the previously studied STFs of various diameter silica particles dispersed, mostly of [BMIm][BF_4_] fluid media (except for one of PEG). A clear negative relativity is observed between the maximum known particle fraction and dimension before transition to solidity. A fluid state of the suspensions is kept for the particle fraction lower than the threshold of dynamic arrest. A ceiling amount below 60 wt.% in Figure 2b is approached when increasing the particle size. This can be explained by a liquid‐lubricated silica particle of similar magnitude thickness, at least not proportional to its diameter. The remaining radius to RCP & DST onset has increased when decreasing the particle radius. Accounting for the permissible higher content of larger radius (< 15 % extra room), it is understandable that the presence of a lubrication layer greater than 5 nm is slightly correlated positively with particle radius, though less than proportionally. This is likely due to the reduced curvature of larger particles, which affects the local geometry.

Interestingly, glass transition also occurs beyond 16 wt.% sometimes (frequently when beyond 18 wt.%), with a higher likelihood of forming a solid with observable yield stress (suggesting a jammed state) at 20 wt.% as a reliable threshold during fabrication. This uncovers the interference of particles at this fraction. An analogous case in previous studies of the dynamic arrest of another colloidal dispersion of nano‐silica in n‐tetradecane witnessed a gelation transition of volume fraction of 15% [46]. The thickness Δ of the liquid medium coated around silica spheres in the adhesive hard sphere (AHS) model can be analogized, whereas the lubrication layer acts as a repulsive interaction in ILSTF colloid dispersion instead. Herein, it is supposed to be ‘arms’ that extend the repulsive range originating from the particle surface.

Based on the finite yield stress threshold of 20 wt.%, we align this threshold to the assumed jammed state near the random close packing (RCP) of static condition [47]. Without shear driven as in rheology, the repulsion‐induced mechanical response is easily attained with slight deformation, acting like a solid. Referring to the previous simulation result of hard spheres without sliding and rolling friction, the volume fraction of isotropic jamming ϕ_*J*_(µ_*s*_ = µ_*r*_ = 0) ≈  0.648 (µ_*s*_: sliding friction coefficient; µ_*r*_: rolling friction coefficient) [44]. In this case, the structural IL lubrication layer thickness is estimated to be  *t*_*lub*2_ = 4.52 nm (Figure 2a) around silica nanospheres of mean radius 6.04 nm. Similarly, when combining the previous rheology results with the commonly found ϕ_*J*_ ≈ 0.56 [10] (of the jamming condition attained via pure shear‐driven, approximate to the ϕ_*J*_(µ_*s*_ = ∞,   µ_*r*_ = 0) = 0.57, supposing no sliding friction but free rolling [44]), we obtain another similar  *t*_*lub*3_ = 4.87 nm (detailed derivation in Section S2). The proof of extension of the particle surface interaction lies in the previous exploration of the interactions in IL (especially [BMIm][BF_4_])—silica system. According to the surface force apparatus (SFA) characterization results (Figure 2c), the typical oscillation‐mode solvation force is evidenced by an onset repulse range of the surface separation *D* ∼ 6.9 nm and several oscillation periodicities (an ion pair dimension) [48]. An augmented interaction originates from the hydrogen bonding between the surface silanol groups and the anion [BF_4_]^−^ in the current ILSTF system. Therefore, the effective interaction range of the two closest nanospheres shifts wider than the case in Figure 2c,d, for example, *D* ∼ 2 *t_lub_* (near 10 nm).

In addition, the equivalent normal stress level and the increasing mode of stress over distance are of major concern as well. As schematically depicted in Figure 2d, the equivalent stress can be derived from the SFA data in the expression below:

(1) $$\sigma_{n}=\frac{F_{N}}{S_{eff}}∼\frac{F_{N}/R}{\pi D}$$

wherein *S_eff_* denotes the effective opposite area of two vertically approached column surfaces.

According to the exponential decay fitting of equivalent stress derivation (Figure 2c,d), the normal stress crosses 2 magnitudes, from 0.032 MPa (*D* ∼ 6.9 nm) to 4.23 MPa (*D* ∼ 1.84 nm), governed by the repulsive structural solvation force of finite IL layers during the driven approach. Comparable stress levels could be achieved when forced‐lubricated nanospheres are further driven into closer contact. This undoubtedly paves the way for jamming dynamics at > 0.1 MPa stress level.

### 2.3 Dynamic Jamming Model

In the transient impact behavior of the ILSTF, the previous shear thickening regime no longer applies. A cylindrical impactor, released in free fall, attains an initial velocity within 2–6 m/s before striking the ILSTF pool surface (initial free surface taken as z = 0; positive z defined downward, detailed setup shown in Section S5). The pool has a much larger diameter and finite depth for the impact case. Upon penetration to a slight degree, the column is rapidly decelerated by the fluid, transferring substantial momentum to the surrounding region and initiating jamming front propagation. As verified in Section S7, only quasi‐static constant loading rate < 10 mm/s has triggered strong shear thickening to marginal jamming in ILSTF (16 wt.%). An impact scenario similar to that in previous work [49] demonstrated that a velocity of 1.5 m/s (E < 10 mJ) is sufficient to trigger jamming, as evidenced by the impactor's bounce upon entering an ILSTF pool of sufficient depth. Impact‐induced jamming or even solidification occurs in this dynamic situation, which is deemed to be a type of isotropic jamming. The IANS model [19] is thus borrowed as the ILSTF direct impact framework of jamming front propagation, with an updated truncated oval‐like added mass geometry [20] referred to as formulas (2) & (3) (detailed derivation of added mass expression attached in Section S8). Formula (2) describes the momentum transfer during the added mass zone propagation just as the penetrating column, and formula (3) depicts the bell‐shaped added mass as a function of penetration depth before the solid plug touches the solid boundary.

(2) $$\left(m_{rod}+m_{a}\left(t\right)\right)a_{rod}\left(t\right)=-\frac{dm_{a}\left(t\right)}{dt}v_{rod}\left(t\right)+F_{ext}\left(t\right)$$

(3) $$m_{a}\left(t\right)=f_{bell}\left(\rho ,k,C,r_{rod},z\left(t\right)\right)$$

wherein

*m_rod_*: the rod mass; *m_a_* (*t*): the added mass in ILSTF; *a_rod_* (*t*): rod acceleration; *v_rod_* (*t*): rod velocity; *F_ext_* (*t*): external forces to the system (rod & ILSTF added mass), which is stagewise analyzed, merely comprised of the gravitational force and the buoyance force before considering direct boundary interaction from stage II; *k*: front propagation coefficient; *C*: added mass coefficient; *r_rod_*: the rod radius, *z*(*t*): rod penetration depth (below the surface). Therefore, the impact process before the jamming front approaches the boundary can be described (detailed data capture is described in Materials and Methods).

**FIGURE 3:**
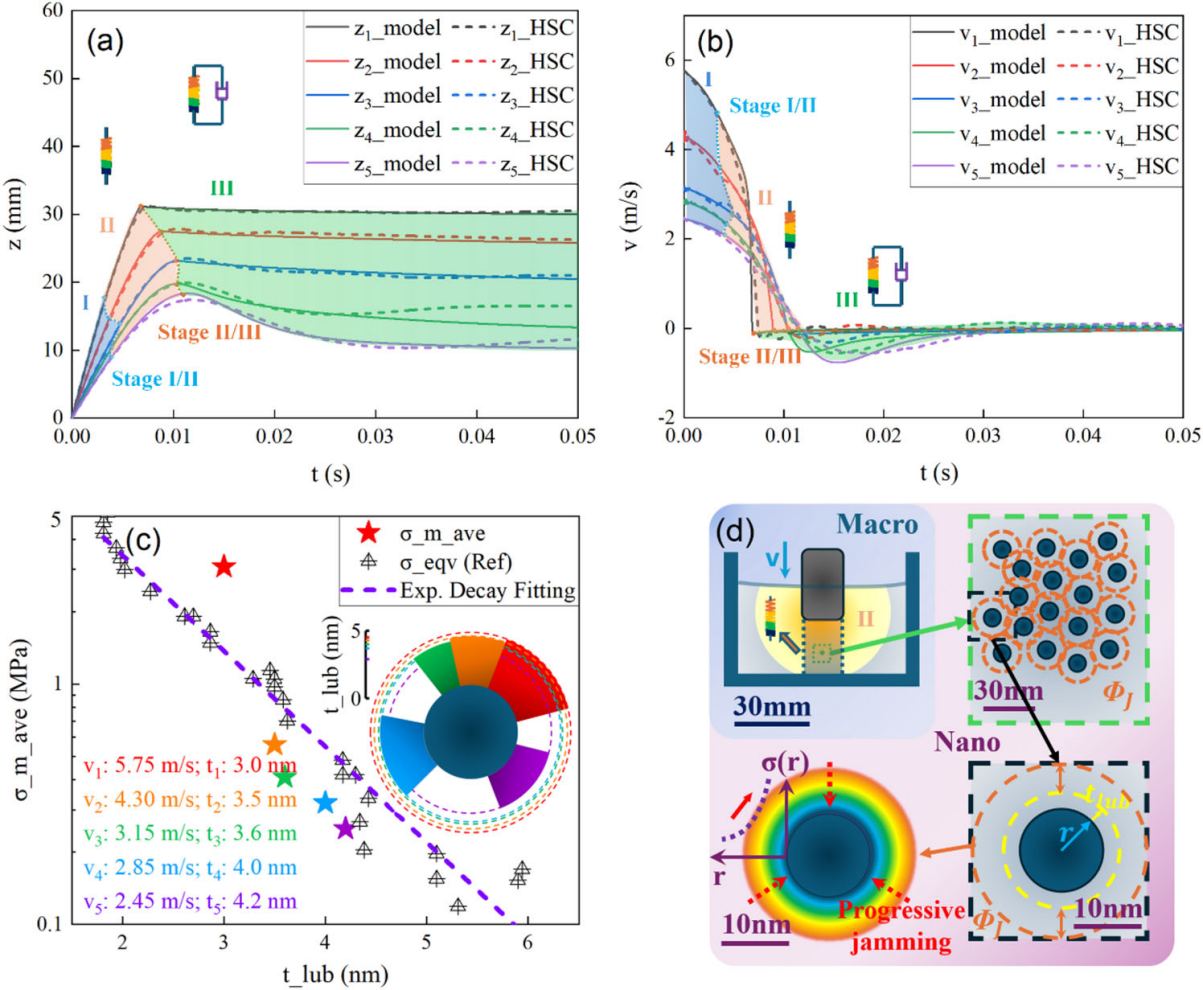
The Modified Added Mass Model fitting and progressive jamming in ILSTF impact test. (a) The Modified Added Mass Model fitting of column z‐t & (b) v‐t plot of various initial impactor velocities upon ILSTF surface penetration, derived from instant column force and verified by scattered points from high‐speed camera (HSC) calibration. (c) Thinner lubrication layer thickness corresponding to the maximum average stress (${\bar{\sigma }}_{m}$, i.e., σ_m_ave) of the jammed ILSTF near the impactor bottom, induced by increasing initial impact velocity. Inset: polar sector diagram illustrating relative lubrication thickness (*t_lub_*) for each velocity; sector, and star colors match those of *v*_1_ ∼ *v*_5_ and the star colors of ${\bar{\sigma }}_{m}$ in subfigure c. (d) Schematic of the Modified Added Mass Model (jamming with lubrication) under scaled‐down dimensions. The inward blue shift of the IL‐lubricated sphere reflects higher stress levels, with the outer layer squeezed away during jamming.

To begin, when the imaginary jamming front propagates freely before meeting the boundary, the classical IANS model is obeyed, or the external force merely includes the gravitational force of the impactor and the buoyancy force of the immersed part. The jammed zone expressed in Equation (3) of the propagating front assumes extra transient momentum absorption and conducts downward, matching stage I in Figure 3a,b. Two critical parameters *k* and *C* are fitted based on the minimized deviation criterion as detailed in Section S9. The instantaneous base force‐time data are provided in Section S10.

Then, based on the optimal fitting, we find a decreasing *k* over initial velocity from 2.7 to 1.0, corresponding to the initial velocity 2.45–5.75 m/s as listed in Table 2. This illustrates a reduced lubrication layer under conditions of increased impact velocity (${\bar{t}}_{lub}$, in Table 2). A physical picture can be described as follows: an enhanced stress level triggered by a more severe direct impact onto the ILSTF system squeezes away the IL of the outer lubrication layer of a static or lower stress level. This process mimicking ‘peeling an onion’ (as shown in Figure 3c,d, a higher stress state in the inner layer of lubrication IL layer) can be termed as a progressive jamming process at a stress level exceeding ∼ 0.1 MPa magnitude for the system, as also found in our previous work [49], corresponding to the referred SFA characterization result of outer layer IL in Figure 2d.

**TABLE 2:** Model parameters and the derived values of various initial velocities.

*v_i_* (m · s^−1^) ^a^ ^)^	*v*_1_	*v*_2_	*v*_3_	*v*_4_	*v*_5_
	5.75	4.30	3.15	2.85	2.45
*C*	0.3				
*k*	1.0	1.4	1.5	2.2	2.7
${\bar{t}}_{lub}$(nm) ^b^ ^)^	3.0	3.5	3.6	4.0	4.2
*K*_0_	2.0	1.5	1.2	0.5	0.5
*K*_1_	1.0	1.8	2.7	3.2	3.5
${\bar{\sigma }}_{m}$(MPa) ^c^ ^)^	3.08	0.56	0.41	0.32	0.25

^a)^ Initial velocity of the impactor upon touching the fluid surface (defined as z = 0).

^b)^ Equivalent thickness of an imaginary lubricated sphere during jamming propagation. Derivation process is elaborated in Section S11.

^c)^ Maximum average normal stress. The derivation process is also elaborated in Section S11.

Subsequently, the jamming front propagates to the solid boundary of the ILSTF holder. The transmitted force to the boundary can address the counter‐intuitive problem of diverging added mass in the Supporting Information of the previous jamming dynamic exploration [19]. Here we go further, building a quantitative model of ILSTF when approaching the solid boundary of much higher velocity and more physically articulated than the prior study of the dynamic constitutional model of water cornstarch dispersion [21]. An imaginary non‐linear spring is added to the external force term of the *F_ext_* (*t*) in (2) of the format described in (4), explaining the direct force transmission between the solid base and the impactor through the spanned jammed zone with further compression.

(4) $$\Delta F_{N}=K_{0}\left\{\exp\left[\frac{K_{1}\left(z_{max}-z_{0}\right)}{z_{max}-z}\right]-\exp\left(K_{1}\right)\right\}$$

wherein

Δ*F_N_*: imaginary spring force; *K*_0_: non‐linear spring response magnitude; *K*_1_: dimensionless non‐linear spring constant; *z_max_*: the maximum depth in the ILSTF pool; *z*_0_: the impactor depth when the imaginary jamming front touches the solid boundary.

As demonstrated by the fitting results in Figure 3a,b, a good agreement is observed for Stage II. *K*_0_ ranges from 2 to 0.5 N (average stress ∼7.8 kPa to ∼2.0 kPa, when normalized by the column cross‐sectional area), with optimal fitting of K_1 from 1.0 to 3.5 (detailed *K*_0_‐value and *K*_1_‐value of arious impact velocities are listed in Table 2), based on the fixed parameters in stage I. The maximum average normal stress (${\bar{\sigma }}_{m}$) of the jammed ILSTF near the impactor reaches 0.25 ~ 3.08 MPa at maximum (*v*_1_ ∼ *v*_5_ in Table 2), which falls within the reference range of 0.03 ~ 4.23 MPa in Figure 2d. This gradient in stress level aligns with a progressively thinner lubrication layer upon solidification to a higher degree during an impact process. The gradually increasing stress level from red to purple (*v*_1_ ∼ *v*_5_) closely matches the stress range derived from the referenced SFA characterization [46] (the colored stars near the purple dashed line in Figure 3c. This agreement emerges when the imaginary lubrication layer thickness is aligned with the surface separation *D*.

In stage III, following the maximum impact displacement, the impactor reverses direction with a reduced rebound velocity as the initial impact velocity increases. This ‘reversed‐bounce’ behavior reveals that the downward viscous drag is governed by the extent of impact‐induced jamming. Once solidification reaches its highest degree, the impactor appears to become arrested near the bottom surface (*v*_5_ case). Here we summarize the governing equation of stage III below in (5), with *ζ* denoting the structural dragging constant (the dashpot in Figure 3d), and well‐fitted in Figure 3a,b below:

(5) $$\left(m_{a}\left(t\right)+m_{rod}\right)a_{rod}\left(t\right)=-\frac{dm_{a}}{dz}{\left(\frac{dz}{dt}\right)}^{2}-\Delta F_{N}+m_{0}g-F_{buoy}-\zeta \frac{dz}{dt}$$

As schematized in Figure 3d, crossing the macroscale to nanoscale, the spring and dashpot, governed by the progressively squeezed lubricated nanoparticle interaction, are gradually utilized for the three stages during the direct impact process. A three‐stage semi‐physical‐based ILSTF Modified Added Mass impact model is thus constructed, simultaneously unveiling the nano‐level mechanisms corresponding to the previous explanation.

## 3 Conclusion

Herein, we revisit a conventionally studied ionic liquid system from rheology to a novel regime of dynamic jamming. The roles played by the persistent meta‐lubrication layer are uncovered during both shear thickening in pure shear and compression‐induced jamming under normal impact, respectively. The solvation layer‐induced repulsion aids in the presumed glass transition and jammed state of the ILSTF system, as well as in the shear thickening process. It greatly reduces the onset particle fraction of both marginal jamming and glass transition. The dependence of the silica fraction further proves the lubricated nature of the strongly continuous shear‐thickened colloidal dispersion. Constitutive models for the ILSTF during dynamic impact were constructed based on the classical IANS [19] model. Physically based non‐DLVO interactions [50, 51] are taken into account when entering the boundary interaction stage. Remarkably accurate fits to the experimental data are achieved using the proposed model with consistent physical parameters across a range of impact velocities. The parameter dependence investigation further proves the existence of the gradient lubrication layer even during the impact‐activated jamming process. This means the structural repulsive force of the outer layers is unable to withstand the extreme stress (at the MPa level). Therefore, the outer solvation layer is increasingly expelled as the impact intensity rises. The transmitted stress is induced during further compression by the remaining momentum input of the already jammed zone. It corresponds in the magnitude level to the previous results of the nano‐level characterizations (SFA & AFM) [48, 52, 53, 54] of IL repulsive forces in similar interfacial distances. This mechanism unveils the potential of this IL‐based STF for dual‐phase jamming transitioning from IL lubricated to friction‐dominated modes under extreme stress conditions.

Beyond their mechanistic novelty, ILSTFs offer self‐adaptive energy absorption together with extreme temperature and pressure tolerance. The multifunctional nature of ionic liquids further extends their significance, suggesting opportunities in aerospace structures, structural electrolytes for energy storage systems, and other applications where impact protection must coexist with electrochemical or environmental functions. These suggest that ILSTFs are not merely incremental improvements over conventional STFs, but rather a platform for a new class of impact‐absorbing, multifunctional materials. While this study provides a solid foundation, further efforts are needed to reinforce and extend the present progress. From a theoretical perspective, the contribution of hydrogen bonding in ILSTF DST/SJ has been deduced, yet the quantitative experimental verification (like what has been done in a previous study [40]) has laid beyond the scope of current work. It will also be important to investigate ionic liquid chemistries with tunable solvation layers. Additionally, though considering the jamming/solidification with the solid boundary (a finite depth pool), it is still a 2D‐simplified case (rotational symmetry), side boundary, and other boundary that cannot be treated as an infinite plane could change the solid‐fluid interaction situation. From an application‐oriented perspective, our experiments were performed under controlled laboratory conditions, whereas real‐world impact scenarios may involve more complex loading conditions, elevated strain rates, or temperature fluctuations that could alter lubrication dynamics. The long‐term stability or degradation effects are another concern. These findings suggest that future studies should broaden parameter space to encompass more extreme stress and temperature conditions and evaluate long‐term cycling under repeated impacts. Progress in scalable processing and composite integration will also be essential for advancing ILSTFs from proof‐of‐concept to application.

## 4 Experimental Section/Methods

### 4.1 Experimental Design

The study aimed to investigate the behaviors of ionic liquid–based shear thickening fluids (ILSTFs) from static to various dynamic conditions, and how the particle interaction governs the process, from glass transition to shear thickening and jamming. ILSTFs with varying silica mass fractions were fabricated, characterized, and subjected to controlled dynamic experiments to assess rheological response and dynamic impact‐activated jamming behavior. All key parameters and procedural details were pre‐specified to ensure reproducibility.

### 4.2 ILSTF Fabrication

ILSTFs were prepared by dispersing hydrophilic nano‐silica (Evonik Aerosil‐200) into the aprotic ionic liquid 1‐butyl‐3‐methylimidazolium tetrafluoroborate ([BMIm][BF_4_], damas‐beta). Both the silica and ionic liquid were dried in a vacuum oven (YAMATO DP33C) at 105°C for 24 h under 0.1 atm. For each standard 100 g batch, silica (8 ~ 20 g) and [BMIm][BF_4_] (80 ~ 92 g) were mixed with acetone (200 g, Yonghua Chemical Co. Ltd). The mixture was sonicated (SCIENTZ, Ningbo) at 40% amplitude for 6 h until a semi‐transparent fluid formed. The dispersion was then heated on a hot plate at 110 °C for 24 h while a magnet rotor initially rotated at 1 000 rpm; the speed was gradually reduced as viscosity increased during acetone removal. The ILSTF was finally evacuated in the vacuum oven at 105°C for an additional 24 h to remove residual solvent and water, producing the high viscosity shear‐thickening fluid.

### 4.3 Morphology Characterization

Particle size and morphology were verified by TEM (JEM‐2100F, JEOL Ltd., Japan; point resolution 0.25 nm, line resolution 0.10 nm). Additionally, nanosilica dispersions (IL and water) were vitrified and imaged by cryo‐TEM. Glow‐discharged holey carbon grids were loaded with 3 ~ 4 µL sample and plunge‐frozen in liquid ethane (Vitrobot, 4^°^C, 100% RH, blot 3.0 s, wait time 5.0 s). Imaging was performed on a Titan Krios G3 at 300 kV using a direct electron detector under low‐dose conditions. Pixel size was calibrated from the microscope metadata, and ImageJ was used to extract the primary diameters (*D_p_*) and center–center spacings (*R_c_*), from which the lubrication half‐gap was computed as δ = (*R_c_* − *D*)/2.

### 4.4 Rheological Characterization

The rheology of ILSTFs was measured with a TA Discovery HR 30 rheometer equipped with a Peltier plate and 1^°^ cone fixture (truncation gap 25 µm). Flow sweeps were performed over shear rates of 0.01–3000 s^−1^, across a wide temperature range, to capture both continuous and discontinuous shear‐thickening behavior.

### 4.5 Drop Column Impact

ILSTFs were subjected to vertical drop column impacts at multiple free‐fall heights. The vertical displacement of the impactor was captured with a high‐speed camera (i‐SPEED 717S, Tokina ATX‐I 100 mm F2.8 FF MACRO), while the instantaneous force at the base platform was monitored with a KISTLER Type 9217A1 sensor and amplified with a KISTLER Type 5018 charge amplifier, then recorded by a YOKOGAWA DLM3034 oscilloscope. In select experiments, a KISTLER Type 8044 acceleration sensor was installed within the drop column to record impactor acceleration. Impact velocity and displacement were calculated using the momentum conservation principle and verified against camera‐calibrated measurements (Figure 3a,b). The calibration of the vertical displacement of the drop column was achieved via Phantom Camera Control (PCC) software from AMETEK (USA).

### 4.6 Dynamic Light Scattering (DLS)

Dense and dilute dispersions of both ILSTF and nano‐silica in water, and PEG‐silica STF were analyzed using dynamic light scattering (Brookhaven Instruments ZetaPALS).

### 4.7 Atomic Force Microscopy (AFM)

Interparticle forces in ionic liquid were measured in a sphere–sphere configuration on a Bruker (JPK) NanoWizard V BioScience AFM. Hydrophilic silica microspheres (D ≈ 10 µm) were used for both the colloidal probe and the substrate: a single sphere was attached to a TESPG‐V2 cantilever, and a second sphere was rigidly fixed on a glass slide. The liquid cell was filled with [BMIM][BF_4_]. Force—distance curves were collected by approach/retract cycles with peak loads from 30 to 1 µN. The cantilever spring constant and deflection sensitivity were calibrated before the test. Raw deflection‐displacement data were converted to force ‐ separation using JPK SPM Data Processing for further analysis in Section S12.

### 4.8 Statistical Analysis

All analyses were conducted using software that allows the results to be independently reproduced from the original data. The size distribution of the nanosilica was determined via TEM characterization. Using calibrated pixel sizes, we quantified N_1_ = 768 primary particles, yielding a diameter distribution with mean ± SD = 12.08 ± 2.77 nm. Independently, calibrated cryo‐TEM measurements of N_2_ = 251 center–center spacings produced a mean ± SD = 21.62 ± 2.80 nm from the Cryo‐TEM calibration. The corresponding distributions are shown in Figure 2a, a representative micrograph is provided in Section S4, and acquisition/processing details were documented in the technical file [55]. The fitting performance of the Added Mass Model was quantified using the coefficient of determination *R* ^2^ and an effective adjusted *R* ^2^ that corrects for temporal autocorrelation in the residuals (via an effective sample‐size estimate). Local robustness of the fitted parameters was assessed by recomputing the effective adjusted *R* ^2^ after systematic ± 10%–30% perturbations of the major parameter group (*k*, *K*_0_, *K*_1_). All model fitting and statistical analyses were implemented in MATLAB (MathWorks, R2021). Detailed statistical analysis is elaborated in Section S9.

### 4.9 Symbols and Abbreviations

For ease of reading, all abbreviations and symbols used in this work are collected in Section S13 (Table S5), with different groups separated by solid horizontal lines.

See Supporting Information for the Parameter List, Derivation of the Lubrication Thicknesses, the Jamming Zone Derivation, and Other Sections, which includes References [56, 57, 58, 59, 60, 61, 62, 63, 64].

## Author Contributions

**Conceptualization**: Y.W. and Y.Y. **Methodology**: Y.W. and Y.Z. **Investigation**: Y.W. and Y.Y. **Visualization**: Y.W. **Supervision**: X.Z. and J.Y. **Writing – original draft**: Y.W. **Writing – review & editing**: Y.W., Y.Z., Q.X., T.X.Y., X.Z., and J.Y.

## Conflicts of Interest

The authors declare no conflicts of interest.

## Supporting information




**Supporting File 1**: advs73617‐sup‐0001‐SuppMat.docx.

**Supporting File 2**: advs73617‐sup‐0002‐VideoS1.mp4.

**Supporting File 3**: advs73617‐sup‐0003‐VideoS2.mp4.

**Supporting File 4**: advs73617‐sup‐0004‐VideoS3.mp4.

## Data Availability

All data needed to evaluate the conclusions in the paper are present in the paper and/or the Supporting Information file. Data are available at Zenodo (DOI:10.5281/zenodo.16275428).
