# Supplementary material for: A Shear Thickening Colloidal Suspension Functioning via Progressive Impact Jamming with Persistent Lubrication Layer
Source: Adv Sci (Weinh). 2026 Jan 20;13(21):e19754. doi: 10.1002/advs.202519754 (PMC13073262; doi:10.1002/advs.202519754)
Supplement: Supplementary file 1 — Supporting File 1: advs73617‐sup‐0001‐SuppMat.docx. [file ADVS-13-e19754-s002.docx]

Supporting Information

A Shear Thickening Colloidal Suspension Functioning via Progressive Impact Jamming with Persistent Lubrication Layer

Yiran Wu, Yifeng Yu, Yiqiu Zhao, Qin Xu, T.X. Yu, Xin Zhang,^*^ Jinglei Yang^*^

Y. Wu, Y. Yu, X. Zhang

Department of Mechanics and Aerospace Engineering, Southern University of Science and Technology, Shenzhen 518055, China

E-mail: zhangx8@sustech.edu.cn

Y. Wu, T.X. Yu, J. Yang

Department of Mechanical and Aerospace Engineering, The Hong Kong University of Science and Technology, Clearwater Bay, Hong Kong SAR

E-mail: maeyang@ust.hk

J. Yang

HKUST Shenzhen–Hong Kong Collaborative Innovation Research Institute, Futian, Shenzhen 518048, China

Y. Zhao, Q. Xu

Department of Physics, The Hong Kong University of Science and Technology, Clearwater Bay, Hong Kong SAR

**This PDF file includes:**

Supporting Text (Section S1 to S10)

Figures S1 to S8 (within SI text)

Tables S1 to S4 (within SI text)

Videos S1 to S3 (representative frame & caption)

References [10,17,20,30,35-37,46-47,56-64] (References are cited according to their numbers in the manuscript reference list)

**Other Supporting Information files for this manuscript include the following:**

Videos S1 to S3

Supporting Text

This Supporting Information file provides additional analyses corresponding to the three main subsections of the Results and Discussion, presented in the order in which they appear therein. Corresponding to Section 2.1 of the main text, we present extended rheological characterizations (shear-thickening hysteresis & shear reversal), the derivation of the lubrication layer thickness, and Mooney model fitting. Second, we first provide morphology characterization of the raw silica nanoparticles by TEM and the nanosilica dispersion state in water and in [BMIm][BF_4_] by Cryo-TEM. We then summarize the fundamental ILSTF parameters, the impact test configuration, and the particle-size dependence of the maximum permissible silica volume fraction in [BMIm][BF_4_]. Furthermore, atomic force microscopy (AFM) is employed to provide further characterization of IL solvation layer between silica particles (Section 2.2). Third, we provide supporting evidence for the shear-thickening-to-jamming transition at low loading rates, an analytical description of the oval-shaped jammed zone, details of the optimized added mass model fitting, including statistical analysis, and model values derivation. (Section 2.3). Additionally, symbols and abbreviations are summarized and listed in an independent section.

S1 Supporting Rheological Analysis: Shear Thickening Hysteresis and Shear Reversal

Flow instability and structural heterogeneity are usually directly linked to the time-dependent behavior in the shear thickening phenomenon. This history-dependent hysteresis is observed due to the different particle spatial structures formed in ascending and descending flow modes.^[56]^ Dynamic structures of particle clusters are formed into metastable states during the process, due in most cases to either inertia or friction.^[57]^ Recent studies have unveiled the relationship between interparticle frictional contact and hysteresis behavior even for colloidal suspensions. ^[58]^

**Figure S1.** Shear thickening hysteresis of ILSTF.

a) A typical shear-thickening hysteresis of ILSTF after 1^st^ experimental cycle and pre-shear. The increasing shear rate (black lines & solid square symbols) and decreasing shear rate (dark blue segments & solid round symbols) curves are in the shear rate range 0.01 ~ 100 s^-1^. b) The first and second shear cycle (three stages: pre-shear, increasing shear rate, decreasing shear rate in sequence) of the ascending and descending flow rates. Figure S1 a & b were both obtained using the ILSTF (16 wt%) (two distinct samples of the same parameter) within a gap of 0.25 mm and a rough boundary surface.

For the current well-dispersed ILSTF of critical silica fraction (16 wt%), a minor yet observable hysteresis is evident in Figure S1A above. Referring to prior studies,^[58]^ a slightly frictional interaction is hypothesized to play a minor role during the ILSTF shear thickening, but the major contribution can be attributed to a hydrodynamic-like interaction that provides relatively fast recovery of the particle clusters. As verified in Supporting Videos S1–S3, which provide synchronized in-situ side-view observations aligned with rheological measurements, the descending flow induces early structural relaxation at higher shear rates for the already formed metastructures, resulting in the redistribution of particles within the medium. Besides, no clear wall-slip in the Videos occurs in the various conditions (t = 1 mm & 0.25 mm, rough & smooth surface) are observed which further bolster the shear thickening saturation of the innate material property of ILSTF instead of structural behavior.

Interestingly, before entering a steady state after the first loading cycle, where a minor hysteresis is reproducible (similar for two distinct samples, as can be compared between Figure S1a & cycle 2 in Figure S1b), compression-induced jamming originated from extra samples placed between the plates of a fixed gap distance. Normal stress exceeding 80 kPa is generated during compression of the jammed colloidial dispersion toward the set gap. A characteristic yield phenomenon emerged from an over 200 Pa shear stress plateau (as shown in Figure S1b) upon the shear start of low shear rate (0.01 s^-1^), even after a pre-shear stage (0.1 s^-1^ for 10 s). After that, it entered the shear-thickened state at a much lower critical shear rate, even near 0.1 s^-1^, reaching a near 6 kPa shear stress before structural breakdown. This initial shear cycle exhibited a pronounced hysteresis, highlighting the ILSTF’s capacity to enter a highly frictional regime under confinement-induced jamming conditions. But this is not triggered by the pure shear loading of reproducible rheological behavior.

As an alternative approach to investigate the rheological mechanisms of STFs, hydrodynamic and contact contributions can be effectively distinguished using shear reversal tests, as demonstrated in a previous study on colloidal suspensions.^[59]^ To further explore the shear thickening nature of the noninertial ILSTF system and elucidate the role played by both mechanisms in ILSTF, a shear reversal test was conducted on ILSTF at the saturation point (ILSTF 16). As illustrated in **Figure S2**a & b, after a pre-shear of 0.1 s^-1^ for 10 s, a shear loading of various magnitudes at constant rate is applied, spanning the entire shear thickening regime of ILSTF (16 wt%). This loading step of 10 s length is followed by an abrupt reversed shear direction of equal magnitude and duration as the last step in each load cycle. The shear rate/strain-time plot verifies the accuracy of the motor control under the current data capture frequency (10 Hz), especially near the reverse direction time spot.

**Figure S2.** Time-resolved loading profiles and rheological responses during shear reversal tests. a) Shear strain, b) shear rate, c) shear stress, and d) viscosity were recorded in real-time at 10 Hz under abrupt reversal of shear rate - from negative to positive - at magnitudes of 1, 5, 10, and 50 s^-1^. Stress levels for each shear step are annotated alongside the corresponding curves. For clarity, low-magnitude shear stress responses are magnified in the bottom-right inset of panel c. At t = 10 s, a vertically dotted cyan line is marked at the shear reversal time spot.

It is illustrated that hydrodynamic and frictional contact correspond to two distinct modes of response upon shear reversal.^[59]^ The sudden loss (‘1 to 0’ mode) of frictional response and symmetric sign-reversing (‘1 to -1’ mode) response of hydrodynamic interaction both contribute during the ILSTF shear thickening process, according to Figure S2c & d. The rheology setup is the same as that of Figure S1a. However, upon a closer look, the detailed mechanism is highly rate dependent. Before and near the shear thickening onset rate (1 & 5 s^-1^) of shear stress below 10 Pa, it appears both mechanisms account for much. Within the shear thickening transition, at the shear rate 10 s^-1^, the contact-like interaction dominates, in contrast to the hydrodynamic-like dominating case above the shear thickening rate (50 s^-1^). This is counterintuitive, considering the normal hydrodynamic to frictional transition instead of the reversed sequence. However, this is rationalized within the IL-lubricated regime. At lower shear rate, the outer ionic layers around the particle of weaker confinement and transient bonding can be easily ruptured yet built up slowly, resulting in a drop in interparticle contact-like forces. But above the shear-thickening rate, the ionic layer has already formed a more robust force network of slight compression, if not yet shear-jammed (shear stress near 4 kPa). The lubrication layer-governed state displays an instantaneous elastic response resembling the behavior of hydrodynamic interactions, which does not exhibit a significant stress drop.

These additional rheology explorations reveal the following physical picture of the ILSTF shear thickening: a hydrodynamically dominated thickening transition of slightly friction-like interaction, particularly below the fully thickened rate. However, a highly frictional regime is accessible for the ILSTF system of compression-induced jamming conditions of much higher stress magnitude.

S2 Derivation of the Ionic Liquid Lubrication Layer Thickness

In the body text, we obtain three estimations of the lubrication layer thickness of IL around nanosilica. The values are correspondingly$t_{lub1}$ = 4.60 nm,$t_{lub2}$ = 4.53 nm, and$t_{lub3}$ = 4.87 nm. The equations used for the derivation, along with the underlying assumptions of the estimations, are provided below.

| $\Phi_{V}=\frac{\Phi_{W}/\rho_{SiO_{2}}}{\Phi_{W}/\rho_{SiO_{2}}+(1-\Phi_{W})/\rho_{IL}}$ | (S1) |
| --- | --- |
|  |  |
|  | (S2) |

wherein

$\phi_{V}$ is the derived silica volume fraction based on the silica weight fraction $\phi_{w}$, $t_{lub}$ is the derived lubrication layer from the actual silica volume fraction $\phi_{V}$ and the referred volume fraction of the lubricated nanoparticles $\phi_{Ref}$ to be aligned to the previous.

***Assumption 1:***

The shear-thickening saturation point (obtained in the rheology test result, Φ_w1_ = 16 %) aligns with the critical volume fraction (Φ_ref1_ = 52 %) where the dispersion reaches the shear jamming point.

***Assumption 2:***

Jammed point (the jammed transition occurs of an observable positive yield stress phenomenon during massive fabrication, Φ_w2_ = 20 %) aligns with the critical volume fraction (Φ_ref 2_ = 64.8 %) where the jam occurs, assuming the sliding frictional coefficient (μ_s_) and the rotational frictional coefficient (μ_r_) are zero. Based on the alignment and derived lubrication layer thickness, the commonly accepted glass transition occurs near 58 vol % ($\boldsymbol{\varphi}_{\boldsymbol{G}}$ = 0.58), corresponding to 17.9 wt%, which coincidentally matches the regular onset of solidity emergence during fabrication.^[44]^

***Assumption 3:***

The shear-thickening saturation point (Φ_w1_ = 16 %) aligns with the commonly obeyed jamming point (Φ_ref3_ = 56 %, which is near the 57 % of the jamming point of μ_s_ = ∞ & μ_r_ = 0).

S3 Mooney Model Fitting of the ILSTF below Shear Thickening

The well-known Einstein relation depicts the relative viscosity of the infinitely dilute spherical solid particle suspensions (for example, $\Phi_{V}<1\%$) in the following Equation (S3):^[60]^

| $\eta_{r}=\frac{1+\frac{1}{2}\Phi}{1-2\Phi}\approx1+2.5\Phi$ | (S3) |
| --- | --- |

herein

$\eta_{r}=\eta_{suspension}/\eta_{medium}$ is the relative viscosity, $\Phi$ is the volume fraction of the particle in dilute suspension (the original source used $c$, here replaced for consistency).

The Mooney model is subsequently developed to account for finite concentrations of rigid spheres at higher volume fractions, with the original dilute case becoming a special limiting condition of the generalized formulation.^[58]^ This is more suitable for the current case of ILSTF (corresponding silica volume fraction listed in the following Table S1).

| $\eta_{r}=\exp(\frac{2.5\Phi}{1-k_{sc}\Phi})=\exp(\frac{2.5\Phi}{1-\frac{\Phi}{\Phi_{m}}})$ | (S4) |
| --- | --- |

In Equation (S4) above, $\Phi_{m}$ is the reference value of the close-packing volume fraction of the particle in dispersion, inversely proportional to the self-crowding factor $k_{sc}$.

To verify the lubricated nature of the nano-silica in [BMIm][BF_4_], we fit the relative viscosity of the static dispersion of the ILSTF of various silica concentrations without applying shear. We first obtain the baseline viscosity of the dispersion fluid medium [BMIm][BF_4_] of $\eta_{0}$ = 0.0965 Pa · s in rheology test. A good agreement is observed between the $\eta_{r}$ derived from the Mooney model under $\Phi_{m}$ = 0.69, and that of ILSTF dispersions prior to the onset of shear thickening of silica weight fraction below 16 wt%. The $\Phi_{m}$ value lies between $\Phi_{FCC/HCP}$ = 0.74 and $\Phi_{RCP}$ = 0.64, as listed in bold in **Table S1** below and displayed in **Figure S3**. This may be attributed to the distinct lubricated nature of silica in ILSTFs from the rigid-sphere assumption in the Mooney model. Another interesting finding is that, beyond the shear thickening saturation threshold 16 wt%, the deviation of the model and the ILSTFs becomes non-negligible. This further proves its lubrication nature, aligning the 16 wt% dispersion to the near-close packing fractions. This is further elaborated in manuscript section 2.2 and Section S2 above, the derivation of the ionic liquid lubrication layer thickness.

**Table S1. Parameters of the ILSTF Dispersion and Relative Viscosity Fitting (under**$\boldsymbol{t}_{\boldsymbol{l}\boldsymbol{ub}\boldsymbol{1}}\boldsymbol{=}\boldsymbol{4}\boldsymbol{.}\boldsymbol{60} \mathbf{nm}$**)**

| Mass Fraction (%) | Volume Fraction (%) | $\eta_{r}$_model (Mooney) | $\eta_{stable}$ (Pa ∙ s) | $\eta$_max (Pa ∙ s) | ${(\eta_{r})}_{stable}$ | $\eta_{r}$_max |
| --- | --- | --- | --- | --- | --- | --- |
| 8 | 4.6 | **2.66** | 0.27 | 0.67 | **2.81** | 6.99 |
| 10 | 5.8 | **4.27** | 0.43 | 1.79 | **4.42** | 18.5 |
| 12 | 7.0 | **8.50** | 0.82 | 11.02 | **8.51** | 114 |
| 14 | 8.2 | **25.4** | 2.50 | 112.52 | **25.9** | 1.17*10^3^ |
| 16 | 9.5 | 190 | 6.96 | 288.98 | 72.1 | 2.99*10^3^ |
| 18 | 10.8 | 2.58*10^4^ | 3.80 | 188.64 | 39.4 | 1.95*10^3^ |
| 20 | 12.1 | > 10^5^ | 4.88 | 214.03 | 50.6 | 2.22*10^3^ |

**
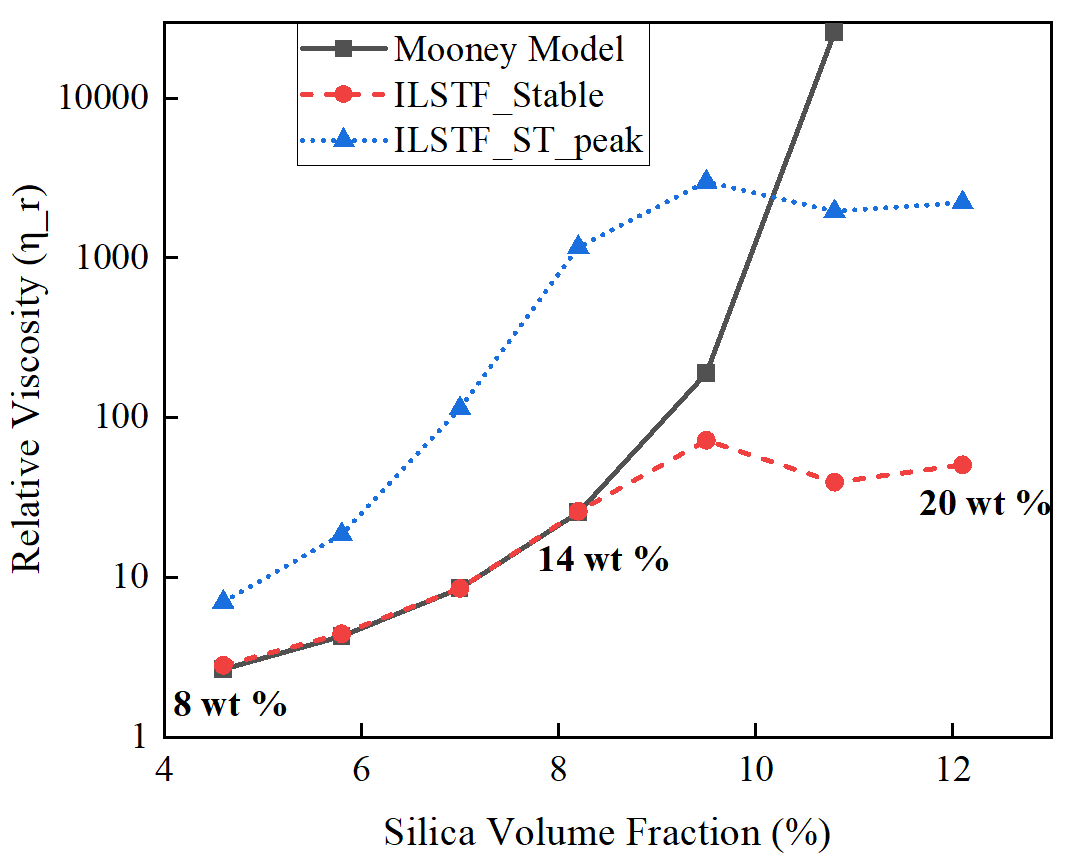
**

**Figure S3.** The Mooney model fitting of the silica dispersion ($\Phi_{m}=0.69$, black solid square symbols & segments), in comparison with the viscosity of ILSTF of various silica volume fractions prior to (red, short-dashed segments & solid round symbols) and right after (blue, short-dotted segments & solid triangular symbols) shear thickening. The points of the lowest fraction, the highest below deviation fraction, and the highest volume fraction below deviation are marked with weight fractions in correspondence.

S4 Nanosilica & ILSTF Morphology

We calibrate the nanosilica size by Transmission Electron Microscopy (TEM) and the center-center spacing between two nanosilicas in ILSTF by Cryo-TEM. To verify the uniform dispersion state of hydrophilic nanosilica in [BMIm][BF_4_] and its gelation-forming tendency in water, the direct observation of both dispersion systems are performed by Cryo-TEM. This flocculation of the nanosilica in water is observed in Figure S4a - c of low particle concentration (< 6 wt%) due to the network-forming nature without effective thick lubrication layer to screen the interparticle hydrogen bonding and van der Waal attraction. In contrast, the nanosilica is well-dispersed in IL as uniformly dispersed primaries (Figure S4d - f) for the structural IL layers lubricateing the surface. The center-center spacing distribution is calibrated to present a mean 21.62 nm with a similar standard deviation to raw particle (2.77 nm $\boldsymbol{\approx}$ 2.80 nm), as demonstrated in manuscript Figure 2a. The lubrication half-gap is then ${\boldsymbol{(}\boldsymbol{t}_{\boldsymbol{lub}}\boldsymbol{)}}_{\boldsymbol{cal}}$ = ($\boldsymbol{R}_{\boldsymbol{c}}$ - $\boldsymbol{d}_{\boldsymbol{p}}$)/2 $\boldsymbol{\approx}$ 4.77 nm, which surprisingly falls within the theoretical estimated range (4.53 nm ~ 4.87 nm). This further supports the aforementioned estimation.


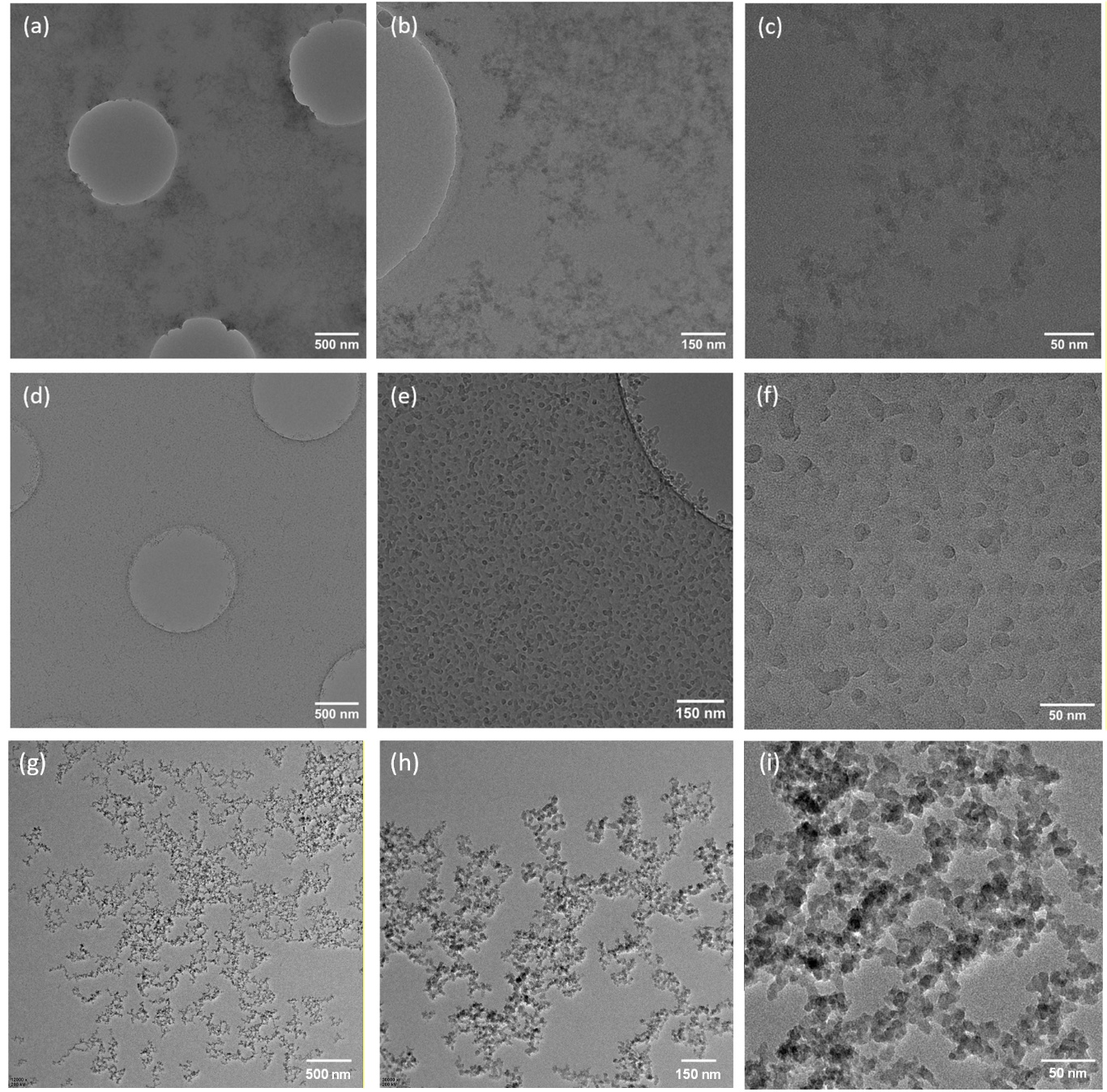


**Figure S4.** Representative Cryo-TEM images of ILSTF (a - f) and TEM images of raw silica nanoparticles (Aerosil-200) (g - i). The average diameter for raw silica is 12.08 nm with a standard deviation of 2.77 nm, based on N_1_ = 768 particles in multiple images, whereas the mean ± SD = 21.62 ± 2.80 nm from the Cryo-TEM calibration of the particle center-center spacings.

S5 Basic Parameters and the Experimental Setup of the Direct Impact Test

**i. Basic Parameters**

**Table S2. Quantities of the physical parameters of ILSTF**

| Parameter | Unit | Value |
| --- | --- | --- |
| ρ_IL^*^ | kg/m^3^ | 1.21 * 10^3^ |
| ρ_SiO_2_^†^ | kg/m^3^ | 2.20 * 10^3^ |
| ρ_ILSTF16^‡^ | kg/m^3^ | 1.29 * 10^3^ |
| d_ave_SiO_2_^§^ | nm | 12.08 |
| d_std_SiO_2_^\|\|^ | nm | 2.77 |
| △(S/M) _SiO_2_^¶^ | m^2^/g | 204 |

* Herein, the IL specifically means [BMIm][BF_4_].

† It specifically means the density of the solid particle of the Aerosil-200 nano-silica, instead of its apparent density.

‡ The density of the ILSTF of silica weight fraction 16 wt%.

§ The average of the statistical result of the Aerosil-200 silica nanoparticles characterized by the TEM photos.

|| The standard deviation of the statistical result of the Aerosil-200 silica nanoparticles characterized by the TEM photos.

¶ The apparent specific surface area derived from the statistical result of the Aerosil-200 silica nanoparticles characterized by the TEM photos. It verifies the referred specific surface area (BET) value of 200 m^2^/g. ^[55]^

**ii. Experimental Setup Parameters**

As demonstrated in **Table S3** & **Figure S5** below, the impact test setup is confirmed by the impactor and ILSTF pool parameters. Additionally, the impact process is monitored by the upper accelerometer and base force sensor.

**Table S3. Parameters and the corresponding details in the experimental setup of the impact test**

| Parameter | Unit | Value | Description |
| --- | --- | --- | --- |
| M_impactor | kg | 0.175/0.197 | Mass of the column-shaped impactor |
| M_ILSTF | kg | 0.31 | ILSTF amount contained in the holder |
| D_impactor | mm | 17 | Diameter of the column-shaped steel impactor |
| D_in | mm | 96.0 | Inner diameter of the ILSTF container |
| z_max | mm | 33.4 | Maximum depth of the ILSTF pool |
| E_min | J | 0.525 | Minimum impact energy |
| E_max | J | 3.26 | Maximum impact energy |


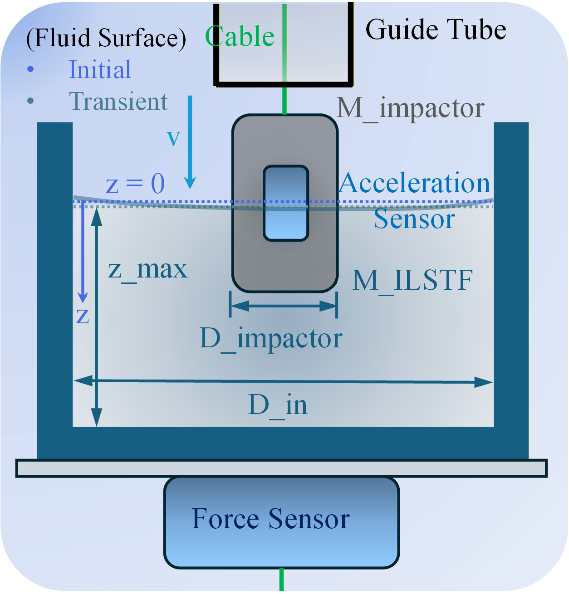


**Figure S5.** Parameters marked in the schematic of the experimental setup in the drop impactor test of the ILSTF pool of shallow depth.

S6 The Size Dependence of the Permissible Silica Fraction in [BMIm][BF_4_]

**Table S4: Silica particles of various dimensions in references^[30,35-37,46,62-64]^**

| Particle Size (nm) | Dispersible Weight Fraction (%) | Dispersible Volume Fraction (%) | Referred Literature | Description |
| --- | --- | --- | --- | --- |
| 500 | 50 | *35.5* | [35] |  |
| 100 | *51* | 40 | [62] |  |
| 55 | 61 | *44* | [63] | Surface fluorocarbon treatment with no hydrogen bonding |
| 50 | 27 | *17* | [36] |  |
| 25 | 39 | *27* | [46] | Octadecanoyl-grafted silica before dynamic arrest |
| 12 | 16 (20^†^) | *9.5 (12*^‡^*)* | Current Work* | Max dispersible before jamming (near glass-transition) |
| 12 | 15 | *8.9* | [30] |  |
| 12 | 14 | *8.2* | [64] |  |
| 12 | 4 | *2.2* | [37] | Deviation from the normal band |

†, ‡ Those marked in brackets are values corresponding to the jammed state, not references. Values before the brackets are glass transition fractions.

The dispersible (weight/volume) fractions are the maximum known dispersible fraction mentioned in the references, approaching but not at the exact upper limit of the corresponding dispersions. The light grey area marks the three cases of larger deviations from the uniform trend. Two of them are due to the surface modification, which causes gelation of a much higher dispersible fraction instead of shear thickening starting at a larger interparticle gap. For the current work, we use the optimized shear-thickening fraction (16 wt%) instead of the maximum permissible dispersion fraction (20 wt%). At 20 wt% the material sometimes behaves as a seemingly non-flowable glass, whereas 16 wt% is the highest loading at which it consistently remains fluid.

S7 Impact-Induced Strong Shear Thickening to Jamming Verification

A strong shear-thickening state of the ILSTF is achieved under pure shear loading during rheological testing. The jamming state becomes accessible under high-magnitude compressive stress, as indicated by the appearance of a yield stress. These results confirm the capability of the ILSTF system to transition from shear thickening to jamming. However, the resulting stress state differs fundamentally from that induced by an impactor submerging into an ILSTF pool. Before conducting direct impact experiments—where normal solidification is expected below the impactor—the onset of ILSTF strong shear thickening to jamming is identified.

A similar round compression head is driven downwards via displacement control first, submerging into the ILSTF pool, just like the direct impact test. The fluid is driven downwards and sideways, applying a shear stress at the submerged column side surface for dragging. There may be normal stress vertical to the bottom of the head, but relatively minor before dynamic jamming occurs, for the bottom boundary is relatively far from the head ($h^{'}\gg\delta^{'}$ in the following Equation (S5), expressing the total vertical force of the head).

| $F=\tau S_{side}+\sigma_{z}S_{bottom}=\overline{\eta}\frac{v_{z}}{\delta'}S_{side}+\overline{\eta}\frac{v_{z}}{h'}S_{bottom}$ | (S5) |
| --- | --- |

wherein

$\tau$ is the shear stress at the side surface of the head, $\sigma_{z}$ is the normal stress at the head bottom, $v_{z}$ is the compression loading rate, $\bar{\eta}$ is the characteristic viscosity of the neighbor flow field of the head, $\delta^{'}$ and $h^{'}$ are the characteristic dimension of the side flow and the compression length scale.

**Figure S6.** Strong shear thickening to jamming of ILSTF under quasi-static compression. The force-displacement (z) curves of the constant loading rate compression by the universal test apparatus. a) Force–displacement curves for the rotationally symmetric case (Test S1). The bottom-left inset highlights the low-force region for comparison, while the top-left inset shows a photo of the experimental setup, with a scale bar of 100 mm. b)- d) The 2D - case setup (Test S2, for flow field observation) of the relatively narrow (δ = 3 mm) and wide (δ = 8 mm) gap between the front & back boundary and the compression head before and near the end of the compression displayed, of increasing loading rate 0.5, 2, and 8 mm/s. A single 20 mm scale bar is shown for each subfigure b-d, applied to both the left & right panels.

The above Equation (S5) belongs to the simplified laminar flow regime, which is suitable for the linear F-z relationship witnessed in both S1 cases for lower loading rate, as depicted in **Figure S6**a. The proportional relationship directly indicates that the dominant contribution arises from side shear forces, which increase with penetration depth, in contrast to the bottom-area normal force that may well remain constant regardless of depth. However, above a critical loading rate, a non-linear F-z relationship, which could be well-fitted by the power function witnessed in Figure S6a, was observed from 4 mm/s as a critical loading rate. Considering the S1 case of ignorable boundary effect, the average shear stress of the side surface of the head is 118 Pa. A characteristic length $\delta^{'}\leq$ 2 mm is observed for the lateral side shear flow as 2D-PIV captured. These generate an average viscosity of around $\bar{\eta}\leq$ 473 Pa · s near the head (beyond the yellow dotted line marking a shear-thickening transition medium reference viscosity value in Figure S6d, r is the distance to the head side surface), displaying a strong shear thickening near the lateral head surface. This also matches the ILSTF16 rheology curve at the critical shear thickening rate lower than 1 s^-1^ and peak viscosity within 280 ~ 450 Pa · s. This indicates that a slightly thinner layer of shear-thickened peak viscosity has covered the side surface of the compression head, and within the characteristic dimension of the side flow, the ILSTF has already been shear-thickened for the linear F-z of low loading rate.

Above the critical loading rate, the relatively stable flow field near the side surface of the compression head starts to expand outward during penetration, indicating a stress increase of the fragile state of the ILSTF approaching shear jamming near the head, marking an increasingly affected flow field during gradual compression. The shear thickening viscosity plateau of much higher shear rate could explain the 2.56 kPa shear stress of the S1 rotational case of 4.27 N peak force (the loading rate 8 mm/s, at z = 15.2 mm) when merely attributing this to the side shear as the normal propagation is still minor in the low loading rate. For the 2D case (Test S2) with near-head boundary restriction, starting from the 0.5 mm/s loading rate, the in-gap ILSTF basically maintains its surface level corresponding to a marginally shear-jammed state as visualized in Figure S6b. At a lower rate, the ILSTF in gap is flowable with increasing surface level as progressive compression. However, the inner side of the slightly jammed is driven downwards at a higher loading rate (from 1 mm/s on for narrow gap case S2-2), simultaneously dragging the outer layer downwards, performing a wall-slip behavior (Figure S6c). As the loading rate further increases, the yield stress is finally met, as evident in the yield pattern in Figure S6d.

Quantitative estimations and direct visual observations confirm the occurrence of pronounced shear thickening and the formation of at least a near-jamming fragile state in ILSTF under quasi-static compression loading at rates below 10 mm/s, using a topologically similar compression head in a pool free of boundary confinement. Notably, in narrow-gap configurations, a transition from shear jamming to yielding can also be induced at higher loading rates within the quasi-static regime. These findings establish a mechanistic basis for understanding the subsequent transition from jamming to solidification under dynamic impact conditions, where loading rates of direct impact exceed those of quasi-static tests by over two orders of magnitude.

S8 Jamming Zone Derivation

The jammed zone is assumed to be oval, verified in the previous experiments of the STF in the quasi-2D mode, and in other explorations of various STFs. In this symmetric condition, the added mass zone is extrapolated to 3D as a rotational body of the 2D case in the XOZ plane. Since the penetration of the impactor is unignorable for the relatively high velocity impact of the current case, we further consider the inner jamming column of the penetration route, sharing the common jamming front in the vertical direction, and of the same diameter as the impactor itself in the horizontal plane. Therefore, the added mass is simplified as a combination of the inner column and the radius extension, tantamount to the radius of the self-similar oval of one near-flat end.

**Figure S7.** Jamming front determination. a) The self-similar growing front fitted by cut-head oval shape (referring the points picked from the scattered points at the jamming front of the quasi-2D case,^[20]^ which is similar in shape to other works^[10,17]^ is described in the optimized fitting Equation (S6) below. b) The cutting-head oval shape is normalized by the dimensionless number A, B, and C (defined as normalized front distance B + C = 1), then c) the normalized impactor radius (R_0_) and the impactor penetration depth (Z) is incorporated in the cross-sectional view of the current 3D case. The imaginary jamming fronts are marked yellow in b and c.

| $\frac{X^{2}}{A^{2}}+\frac{(Z-C)^{2}}{B^{2}}=1$ | (S6) |
| --- | --- |

wherein

A = 0.50, B = 0.76, C = 0.24. Then the jamming front extracted from Figure 7a, depicted in Figure 7b and Equation (S6), is modified according to unignorable penetration in the current case, as displayed in Figure 7c as a cross-section of the current case. It is in a simplified version (but with acceptable accuracy, though not mathematically rigorous), which is the impactor radius extrapolation of the previous front in the radial direction. Based on the assumption, the volume of the jamming front-enveloped ILSTF, the added mass, can be expressed in the following equations (S7) - (S10), considering the density of the ILSTF (16 wt%).

The algebraic form of the added mass volume can be expressed as

| $V_{added-mass}=\int_{0}^{(k+1)z} \pi r(z)^{2}dz-\int_{0}^{z} \pi r_{0}^{2}dz=\int_{0}^{(k+1)z} \pi(r_{0}+x(z))^{2}-\pi r_{0}^{2}z=k\pi r_{0}^{2}z+\pi r_{0}\int_{0}^{(k+1)z} 2x(z)dz+\int_{0}^{(k+1)z} \pi x(z)^{2}dz$ | (S7) |
| --- | --- |

Then, according to the numerical integration by MATLAB, the above equals to the form below

| $V_{added-mass}=3.1416kr_{0}^{2}z+1.1284(\left(k+1\right)z)^{2}+0.7476(\left(k+1\right)z)^{3}$ | (S8) |
| --- | --- |

Then the added mass can be derived when multiplying the above (S8) by the ρ_ILSTF16

| $m_{a}=964.4(k+1)^{3}z^{3}+1456r_{0}(k+1)^{2}z^{2}+4053kr_{0}^{2}z$ | (S9) |
| --- | --- |

For the convenience of the substitution of the governing equation in the modified added mass model, the derivative of the added mass of impactor penetration depth z is expressed below

| $\frac{dm_{a}}{dz}=2893(k+1)^{3}z^{2}+2912r_{0}(k+1)^{2}z+4053kr_{0}^{2}$ | (S10) |
| --- | --- |

S9 Dynamic Jamming Model Parameters: Statistical Analysis

**i. Confirmation of the governing parameters** ***k* & *C***

Two critical parameters *k* and *C* are fitted based on the minimized deviation criterion, defined by the average square residual (ASR), as defined in Equation (S11) below.

| $ASR=\frac{\sum_{\text{i}=1}^{n_{\exp\text{rfit}}} (z_{\mathrm{mod}\text{el}}(t_{i})-z_{\exp\text{r}}(t_{i}))^{2}}{n_{\exp\text{rfit}}\sqrt{\sum_{i}^{n_{\exp\text{rfit}}} z_{\mathrm{mod}\text{el}}^{2}(t_{i})\sum_{i}^{n_{\exp rfit}} z_{\exp\text{r}}^{2}(t_{i})}}$ | (S11) |
| --- | --- |

To directly visualize the non-convex nature of the optimized *k* - *C* relationship, a characteristic “*k* - *C* valley” can be observed in Figure S8 for a representative impact case. The *z*-axis is the ASR, corresponding to a well-fitting result, a low deviation of the model from the experiment.


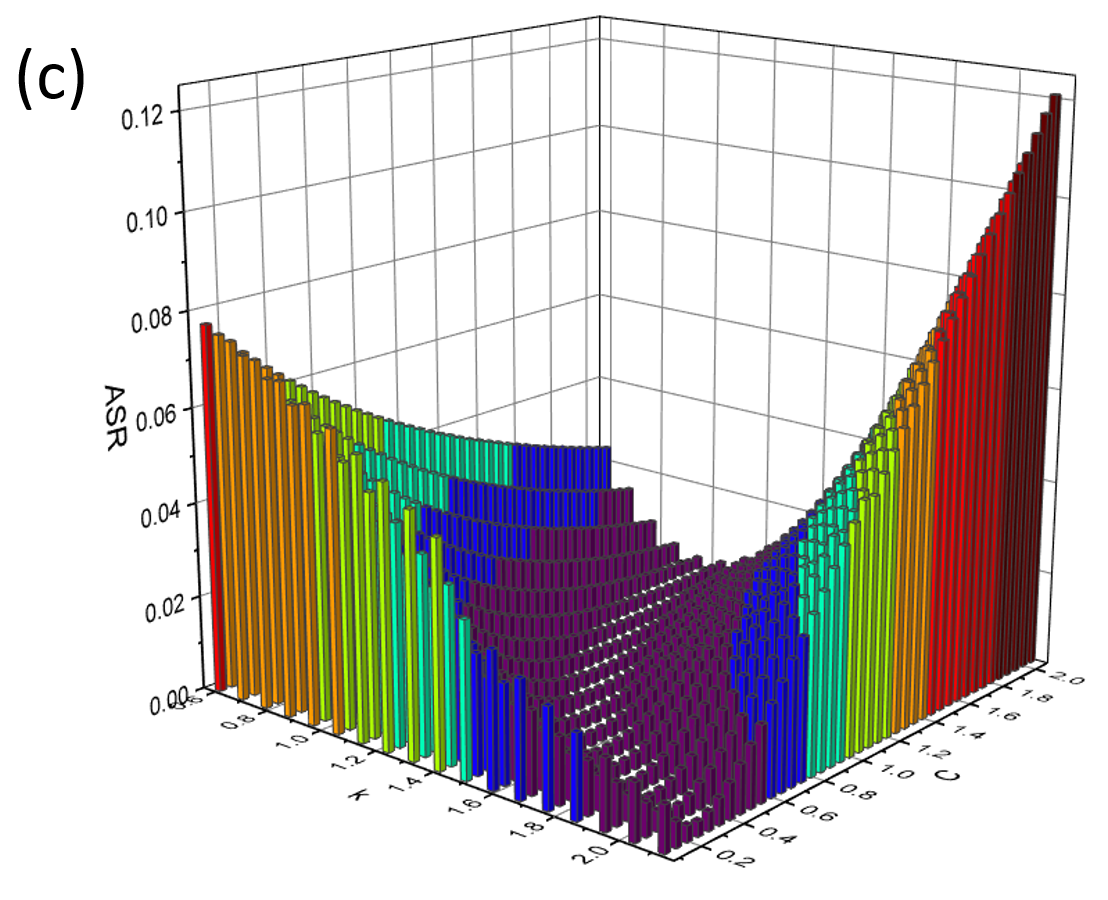


**Figure S8.** Average ASR of the controlling parameters *k* & *C*. It demonstrates a ‘*k* - *C* valley’ of stage I during the impact of a typical impact case.

From the experimental result (stage I) in Figure 3a (manuscript), a perfect alignment is attained utilizing *C* = 0.3 about the velocity dependence, marked by the uniform displacement deviation of another series of large-depth impact experiments. Therefore, the optimized k is confirmed by the intersection of the ‘valley line’ at *C* = 0.3. In this way, the optimized model parameters are determined, as listed in Table I in the manuscript. The derivation process of the equivalent lubrication layer thickness ($\bar{t}_{lub}$) and the maximum average normal stress ($\bar{\sigma}_{m}$) is elaborated in Section S10.

**ⅱ. Model Fitting Evaluation**

To quantitatively assess the quality of the whole model fits, we combined global goodness-of-fit metrics with a residual-based uncertainty analysis for all fitted parameters. The analysis was performed jointly over all trajectories (five initial impact velocities, displacement–time and velocity - time data). All analyses were implemented in MATLAB (MathWorks, R2021).

(1) Goodness of Fit

For each fitted case, we computed the coefficient of determination ($R^{2}$, as defined in Eq. (S12) below)

| $R^{2}=1-\frac{SS_{res}}{SS_{tot}}$ | (S12) |
| --- | --- |

where $SS_{\mathrm{res}}=\sum_{i} (y_{i}-\hat{y}_{i})^{2}$ is the residual sum of squares and $SS_{\mathrm{tot}}=\sum_{i} (y_{i}-y)^{2}$ is the total sum of squares based on all experimental data points $y_{i}$ and model predictions $\hat{y}_{i}$. We additionally report the conventional adjusted $R^{2}$ ($R_{\mathrm{adj}}^{2}$ as defined in Eq. (S13)),

| $R_{adj}^{2}=1-(1-R^{2})\frac{n-1}{n-p-1}$ | (S13) |
| --- | --- |

with $n$ the total number of data points and $p$ the number of fitted parameters. For the optimal parameter vector $\theta_{\text{opt}}$, we obtain $R^{2}\approx R_{\mathrm{adj}}^{2}\approx$ 0.9975, indicating an excellent overall agreement between model and experiment.

(2) Effective Sample Size and Temporal Autocorrelation

Because the impact time series are densely sampled and strongly time-correlated, using the nominal sample size $n$ in $R_{\mathrm{adj}}^{2}$ would overestimate the effective degrees of freedom. We therefore estimated an effective sample size $n_{eff}$ from the residual autocorrelation. Each trajectory contains 3125 sampling points (0 ~ 50 ms). Residuals were computed for the five displacement and five velocity curves, and their autocorrelation functions were averaged to obtain a mean lag-$k$ autocorrelation $\rho_{k}$. Applying a Bartlett-type correction with a cutoff lag $K$ = 16 yielded an effective sample size per trajectory $n_{\mathrm{eff},\mathrm{single}}$ (as depicted in Eq. (S14)), and the total effective sample size across all ten residual series was

| $n_{eff}=\frac{10n}{1+2\sum_{k=1}^{K} (1-\frac{k}{n})\rho_{k}}$ | (S14) |
| --- | --- |

The total effective sample size across all trajectories was taken as $\text{ }n_{\mathrm{eff}}\approx100.6.$ This effective sample size $n_{\mathrm{eff}}$ was used as the degree of freedom (DOF) when estimating the residual variance and constructing confidence intervals. Replacing $n$ by $n_{\mathrm{eff}}$ in the $R_{adj}^{2}$ definition gives an effective adjusted coefficient of determination $(R_{\mathrm{adj}}^{2})_{\mathrm{eff}}$. For the optimal fit we obtain $(R_{\mathrm{adj}}^{2})_{\mathrm{eff}}\approx$ 0.9969. This confirms that the model reproduces the experimental curves with very high fidelity even after correcting for temporal correlations.

3) Local robustness around $\theta_{\text{opt}}$

To probe the robustness of the fitted parameters, we examined how $(R_{\mathrm{adj}}^{2})_{\mathrm{eff}}$ changes when the stage I and stage II parameters are perturbed around $\theta_{\mathrm{opt}}$. For each major parameter group ($k$, $K_{0}$, $K_{1}$), the corresponding five values (for the five impact velocities) were uniformly scaled by factors $\lambda\in${0.7,0.9,1.0,1.1,1.3}, while all other parameters were kept fixed. For each $\lambda$, we recomputed $(R_{\mathrm{adj}}^{2})_{\mathrm{eff}}(\lambda)$ from the full data set. The resulting profiles (Figure S8b) show that for all three parameter groups, changing the parameters by ± 10% around $\theta_{\mathrm{opt}}$ alters $(R_{\mathrm{adj}}^{2})_{\mathrm{eff}}$ by less than ~ 1% relative to its maximum, whereas ± 30% deviations lead to a clear deterioration of the fit quality, most prominently for $K_{1}$. This demonstrates that the optimal parameter set lies in a narrow basin of high fit quality and that the practically relevant uncertainty of $k$, $K_{0}$, and $K_{1}$ is on the order of ± 10%.


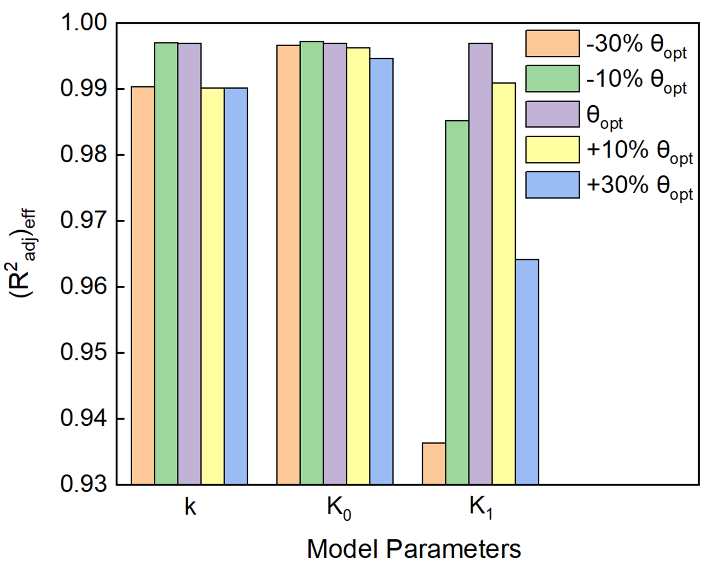


**Figure S9.** Effective adjusted $(R_{\mathrm{adj}}^{2})_{\mathrm{eff}}(\lambda)$ for the parameter groups $k$, $K_{0}$ and $K_{1}$when all five $\lambda$ values (0.7,0.9,1.0,1.1,1.3) (i.e. − 30%, − 10%, optimum, + 10%, + 30%, as the legend displays).

The combined evidence from various $R^{2}$ and the local $(R_{\mathrm{adj}}^{2})_{\mathrm{eff}}$ profiles (Figure S8b) is sufficient to demonstrate both the quality and the robustness of the fitted parameters.

S10 Impact Force: Time Histories

The instant base force - time of various initial impact velocities is displayed in **Figure S10**. An abrupt force increase is witnessed of the highest initial velocity, denoting the contribution by the non-linear spring in stage II. The peak values of the force – time profiles are utilized to generate the maximum average normal stresses $\bar{\sigma}_{m}$ (peak force devided by the impactor bottom area), as listed in manuscript Table 2.


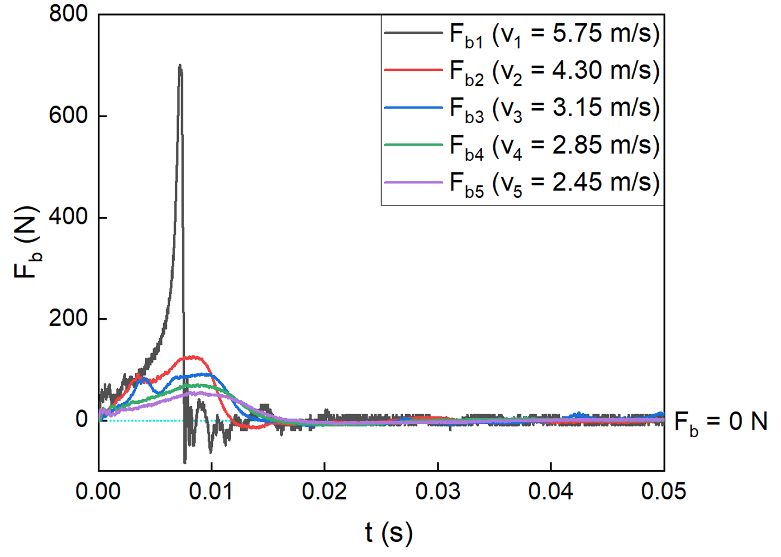


**Figure S10**. Additional base force - time response of various initial velocities.

S11 Derived Values in the Dynamic Jamming Model

Herein, we elaborate on the derivation process of the values, $\bar{t}_{lub}$ and $\bar{\sigma}_{m}$ in Table I (manuscript).

The equivalent lubrication layer thickness $\bar{t}_{lub}$ is calculated based on the relationship between the front propagation ratio *k* and the current particle volume fraction in the suspension, expressed below in Equation (S15)^[20]^.

| $k=\frac{\Phi_{0}}{\Phi_{J}-\Phi_{0}}$ | (S15) |
| --- | --- |

$\phi_{J}$ is set to be the Φ_ref 2_ = 64.8 %, in Section S2 (*Assumption* 2), reflecting the lubrication-dominated nature of the ILSTF. Since the bare particle volume fraction of ILSTF (16 wt%) is 8.10 %, according to Equation (S1). From Equation (S12), we further obtain the $\phi_{0}$ for each *k* value from 0.7 to 2.0 (in Table 1). The equivalent thickness of an imaginary lubricated sphere ($\bar{t}_{lub}$) during jamming propagation is thus derived using Equation (S3).

The maximum average normal stress ($\bar{\sigma}_{m}$) of the jammed ILSTF near the impactor is estimated by the peak instantaneous force of each impact case over the bottom area of the impactor (Table S3), as the bottom jammed region dominates the vertical response in solidification.

**S12 Characterization of Intersilica Force in-Ionic Liquid**

Besides the details mentioned in the Experimental Section/Methods, the sensitivity (14.50 nm/V) and spring constant (7.653 N/m) were calibrated during the test and utilized in data processing. Adhesion force and the Young’s Modulus (Hertz/Sneddon) were obtained in the standard data processing by the JPK SPM software, and the analysis was based on the corrected force - position exported after the processing.

During the test, two 10 μm - diameter silica microspheres were employed, one glued onto a glass slide (**Figure S10**a) and the other attached to the cantilever tip (Figure S10b), and a series of normal approach/retract tests were performed over a wide range of prescribed maximum loads (Figure S10c). Two stages are included in a cycle, approach and retraction, as shown in typical force (F’) - surface separation (D’) curves in Figure S10d. A good repeatability was achieved in 3 consecutive tests for each force magnitude, illustrating the reproducible short-range repulsion. However, the non-linear repulsive relationship (F’ - D’) has induced a force magnitude-dependent equivalent modulus during approach (E_apr), and simultaneously a dependent adhesion force (minimum value F’ < 0 in Figure S10d), as displayed in Figure S10e. Both the unneglectable adhesion force and ultrahigh modulus (exceeding 1 GPa at max) corroborate the local solidification of interparticle confined IL before ‘melting’ at the nano-level, which can also explain the dashpot-like macroscale behavior in stage Ⅲ in the Modified Added Mass Model. The analysis of the force-distance curves reveals a lubrication (solvation) layer thickness $t_{lub}\approx$ 5.5 nm surrounding each 10 μm sphere, persisting over two orders of magnitude in the average equivalent normal stress, as demonstrated by Figure S10f. In view of the cooperative structuring (local force fields superposition, may well be contributed by the surface hydrogen bonding + IL short range ‘structural arms’) and thus enhanced solvent ordering expected for larger particles, a positive correlation between lubrication-layer thickness and particle size is reasonable; accordingly, an increase in thickness from ~ 4 - 5 nm for 12 nm primary particles to around 5.5 nm for 10 μm silica spheres is consistent with this picture.


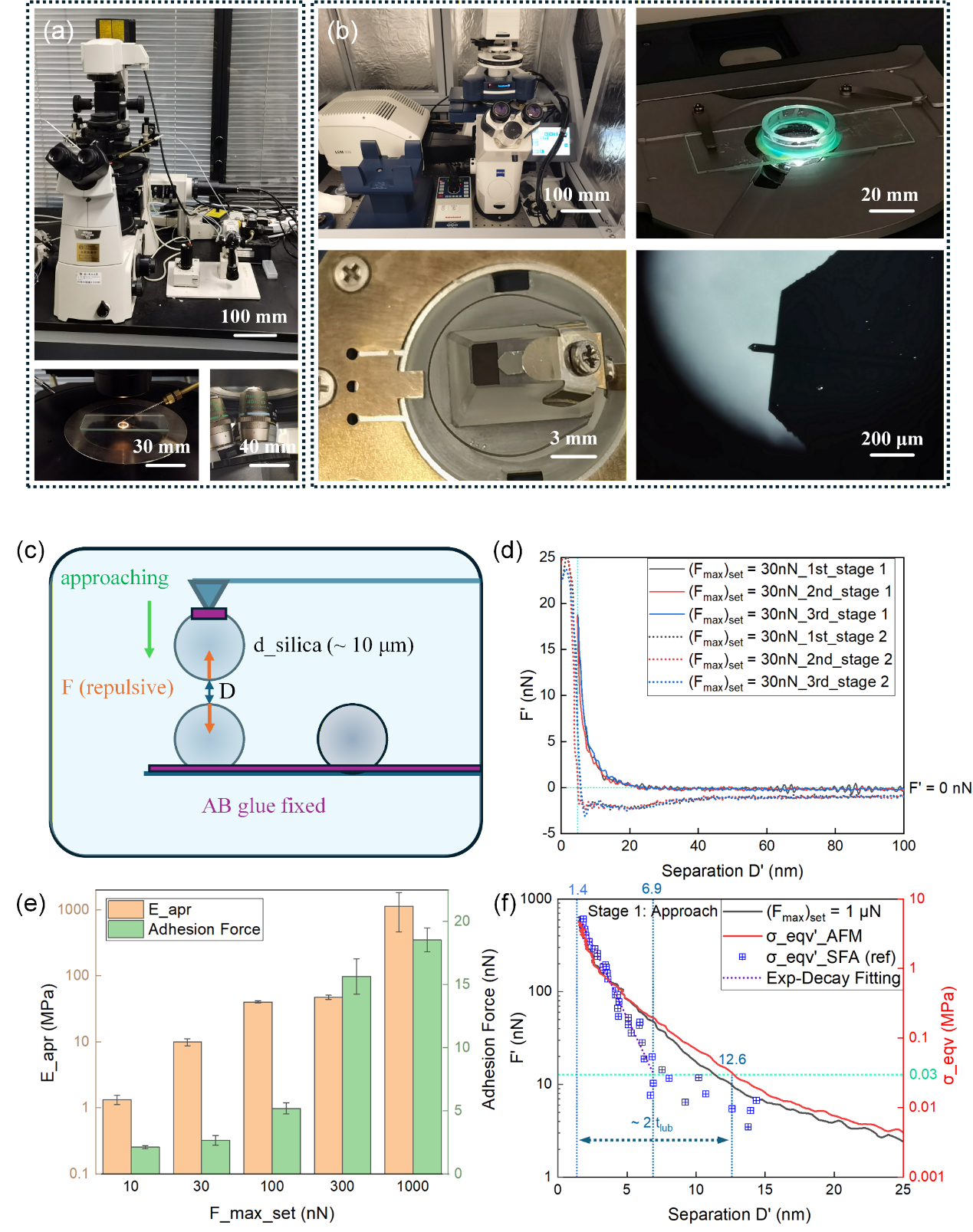


**Figure S10.** Targeted interfacial measurement of the nanoscale lubrication layer surrounding (hydrophilic) silica in IL. a) Sample fabrication assisted by the micromanipulator and microscopy, b) AFM micro-compression set-up (NanoWizard V), showing the AFM platform, an IL-filled sample cell confined by a circular wall, the cantilever holder, and the silica colloidal probe tip under the microscope. c) Schematic of the AFM micro-compression configuration, d) representative force-distance curves for repeated approach-retraction cycles at a preset maximum load ${(F_{max})}_{set}$ = 30 nN, e) elastic modulus of approaching repulsive forces and adhesion forces of various set maximum forces (${(F_{max})}_{set}$, 10 to 1000 nN), f) repulsive force and equivalent average stress proving the IL layer existence.

**S1****3 Symbols and Abbreviations**

For ease of reading, all abbreviations and symbols used in this work are collected in Table S5, with different groups separated by solid horizontal lines.

**Table S5. Summary of symbols and abbreviations used in this study**

| **Abbreviation** | **Description** | |
| --- | --- | --- |
| (IL)STF | (Ionic liquid-based) Shear thickening fluid | |
| IANS | Impact-activated normal solidification | |
| CST | Continuous shear thickening ($\beta<1$) | |
| DST | Discontinuous shear thickening ($\beta>1$) | |
| SJ | Shear jamming | |
| EG/ PEG/ PPG | Ethylene glycol/ polyethylene glycol/ polypropylene glycol | |
| AHS | Adhesive hard sphere | |
| FCC/ HCP/ RCP | Face-centered cubic/ hexagonal close-packed/ random close-packed | |
| SFA | Surface force apparatus | |
| AFM | Atomic force microscopy | |
| ASR | Average squared residual (for model fitting) | |
| DOF | Degree of freeedom | |
| **Symbol** | **Unit** | **Explanation** |
| $\eta$ |  | Viscosity |
| $\eta_{r}$ |  | Relative viscosity ($\eta_{r}= \eta_{suspension}/\eta_{medium}$) |
| $\eta_{stable}$ | Pa · s | Viscosity in low shear rate regime (below the shear thickening onset) of stable dispersions |
| $\eta_{max}$ | Pa · s | Maximum viscosity (over the applied shear rate range) |
| dγ/dt | s^-1^ | Applied shear rate |
| $\beta$ |  | Shear thickening strength ($\beta=\log\left( \Delta\eta\right)/\log\left( \Delta\sigma\right)$) |
| $\tau_{ma}$ | Pa | Maximum allowable shear stress (plateau) |
| ST Ratio |  | Shear thickening ratio (defined by $\eta_{max}/\eta$*_*stable) |
| $\Phi$(or $\Phi_{V}$) |  | Particle volume fraction in dispersion |
| $\Phi_{J}$ |  | Jamming volume fraction |
| $\Phi_{0}$ |  | Raw silica particle volume fraction in ILSTF |
| $\Phi_{m}$ |  | Reference value of the close-packing (maximum) volume fraction in Mooney model |
| $k_{sc}$ |  | Self-crowding factor in Mooney model |
| $\Phi_{w}$ |  | Particle mass fraction in dispersion |
| $\Phi_{w1}$($\Phi_{w2}$) |  | Shear thickening saturation point (16 wt%) (Solidity emergence point (20 wt%)) |
| $\Phi_{ref1}$ |  | Critical volume fraction of shear jamming threshold (0.52) |
| $\Phi_{ref2}$ |  | Critical volume fraction of jam occurence (0.648) |
| $\Phi_{G}$ |  | Volume fraction at the glass transition point (0.58) |
| $\Phi_{ref3}$ |  | Commonly obeyed jamming point |
| $\Phi_{FCC/HCP}$ |  | 0.74 (face-centered cubic/hexagonal close-packing volume fraction) |
| $\Phi_{RCP}$ |  | 0.64 (3D random close-packing volume fraction) |
| $t_{lub}$ | nm | Lubrication layer thickness |
| $D$ | nm | Surface separation (in SFA characterization, between two columns; in ILSTF, between neighboring silica surfaces) |
| $R$(or $d_{p}$) | nm | Radius (or diameter) of the silica nanoparticle |
| $R_{C}$ | nm | Center-center spacing between two neighbor silica nanoparticles |
| $\mu_{s}$ |  | Sliding frictional coefficient |
| $\mu_{r}$ |  | Rotational frictional coefficient |
| $\sigma_{n}$ | MPa | Normal stress (in SFA) |
| $F_{N}$ | N | Normal force (in SFA between columns) |
| *S_eff_* | nm^2^ | Effective opposite area of two vertically approached column surfaces (in SFA) |
| $m_{rod}$ | kg | The rod (impactor) mass |
| $m_{a}\left( t \right)$ | kg | The added mass in ILSTF which varies by time |
| $v_{rod}\left( t \right)$ | m · s^-1^ | Impactor (rod) velocity, as a function of time |
| $a_{rod}\left( t \right)$ | m · s^-2^ | Impactor (rod) acceleration, as a function of time |
| $V_{added-mass}$ | m^3^ | Volume of the added-mass region around the impactor |
| $r_{0}$ | m | Rod (impactor) radius |
| $F_{ext}\left( t \right)$ | N | The external forces to the ‘rod & ILSTF added mass’ system |
| $k$ |  | Front propagation coefficient |
| $C$ |  | The added mass coefficient |
| $r_{rod}$ | m | The rod radius |
| $z\left( t \right)$ | m | The rod penetration depth (below the initial fluid surface) |
| $z_{\mathrm{model}}$ | m | Displacement fitted by the Modified Added Mass Model |
| $z_{\mathrm{expr}}$ | m | Displacement of the impactor obtained from the test |
| $n_{\mathrm{exprfit}}$ | m | Discrete displacement points number used in the Stage I model fitting |
| ${\Delta F}_{N}$ | N | Imaginary spring force (from Stage II on) |
| $K_{0}$ | N · m^-1^ | Non-linear spring response magnitude |
| $K_{1}$ |  | Dimensionless non-linear spring constant |
| $z_{max}$ | m | The maximum depth in the ILSTF pool |
| $z_{0}$ | m | The impactor depth when the imaginary jamming front touches the solid boundary ($z_{0}=z_{max}/(k+1)$) |
| $v_{i} (i=1\sim5)$ | m · s^-1^ | Initial velocity of the rod upon touching the fluid surface |
| $\bar{\sigma}_{m}$($\sigma\_m\_ave$) | MPa | The maximum average normal stress |
| $\bar{t}_{lub}$ | nm | Equivalent thickness of an imaginary lubricated sphere during jamming propagation |
| $F_{buoy}$ | N | The buoyancy force of the fluid on the impactor |
| *ζ* | kg · s^-1^ | The structural dragging constant after solidification |
| $\tau$ | MPa | Shear stress at the side surface of the head (in Quasi-static Compression) |
| $\sigma_{z}$ | MPa | Normal stress at the head bottom |
| $v_{z}$ | m · s^-1^ | Compression loading rate |
| $\bar{\eta}$ | Pa · s | Characteristic viscosity of the neighbor flow field of the head |
| $\delta^{'}$(or $h^{'}$) | mm | Characteristic dimension of the side flow (or the bottom flow upon compression) length scale |
| X |  | x-axis coordinate of dimensionless jamming front edge |
| Z |  | z-axis coordinate of dimensionless jamming front edge |
| A |  | Semi-minor axis dimensionless length of the jamming front oval (along x direction), normalized by the cutting-head major axis length |
| B |  | Semi-major axis dimensionless length (along z direction) |
| C |  | Semi-major axis dimensionless length after cutting head |
| $R^{2}$ |  | Coefficient of determination |
| $y_{i}$ |  | Discrete experimental data points for model fitting evaluation |
| $\hat{y}_{i}$ |  | Model prediction values at corresponding time spots |
| $SS_{\mathrm{res}}$ |  | Residual sum of squares |
| $SS_{\mathrm{tot}}$ |  | Total sum of squares based on experimental data points |
| $R_{\mathrm{adj}}^{2}$ |  | Adjusted coefficient of determination |
| $\theta_{\text{opt}}$ |  | Optimal model parameter vector |
| $n_{eff}$ |  | Effective sample size |
| $\rho_{k}$ |  | mean lag-$k$ autocorrelation based on autocorrelation functions of z-t and v-t curves |
| $(R_{\mathrm{adj}}^{2})_{\mathrm{eff}}$ |  | Effective adjusted coefficient of determination based on effective sample size |
| $\Delta_{j}^{\left( m \right)}$ |  | Perturbed parameter (j th) increment |
| $\lambda$ |  | Scaled factor of perturbed parameter increment normalized by the corresponding optimal model parameter. |
| F’ | nN | Typical force measured at the AFM tip |
| D’ | nm | Surface separation between the colloidal tip and the bottom silica sphere |
| E_apr | MPa | Equivalent modulus during approach (AFM) |


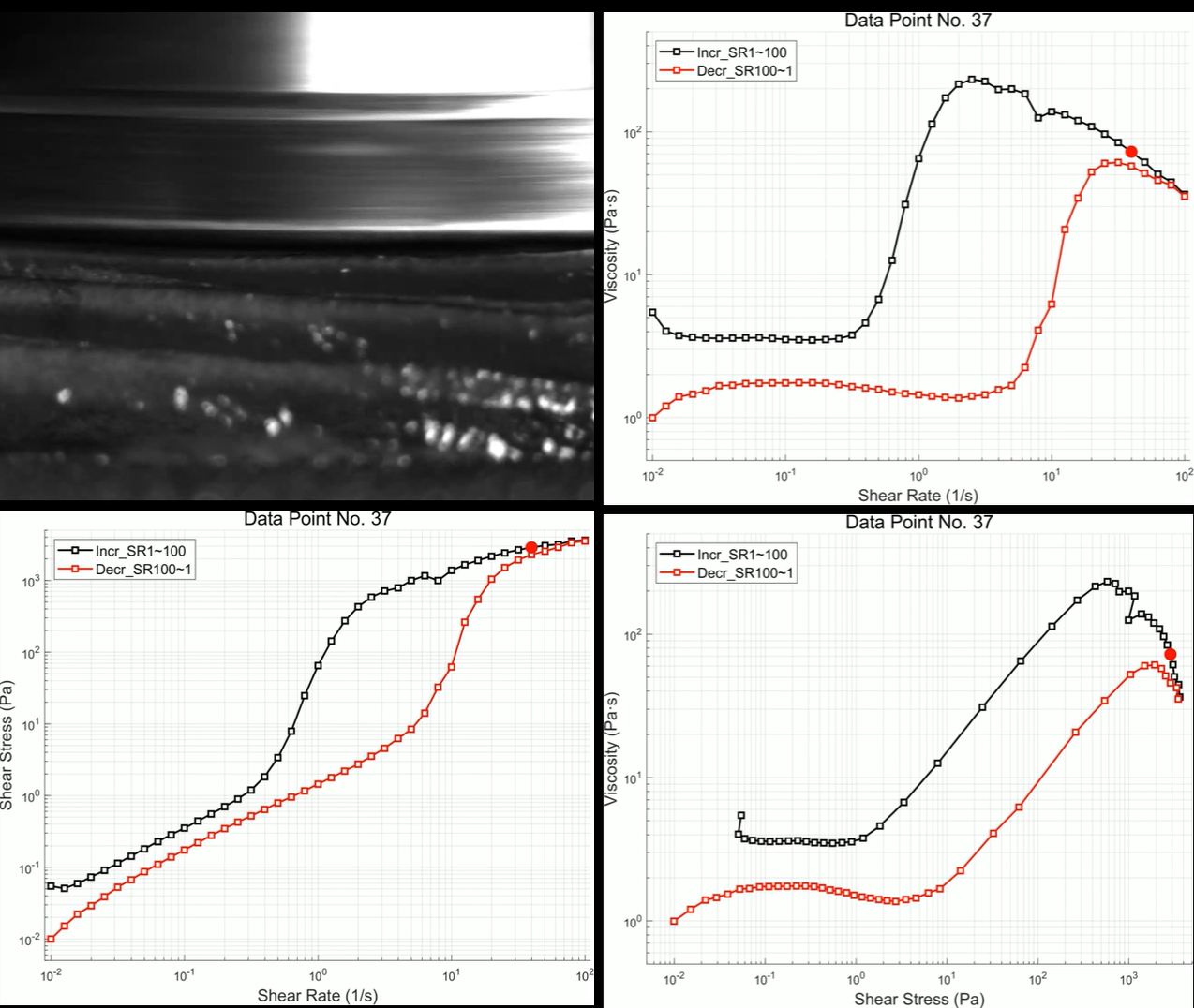


Video S1. Synchronized side-view observations aligned with rheological measurements. Gap distance 1 mm & rough surface at the bottom.


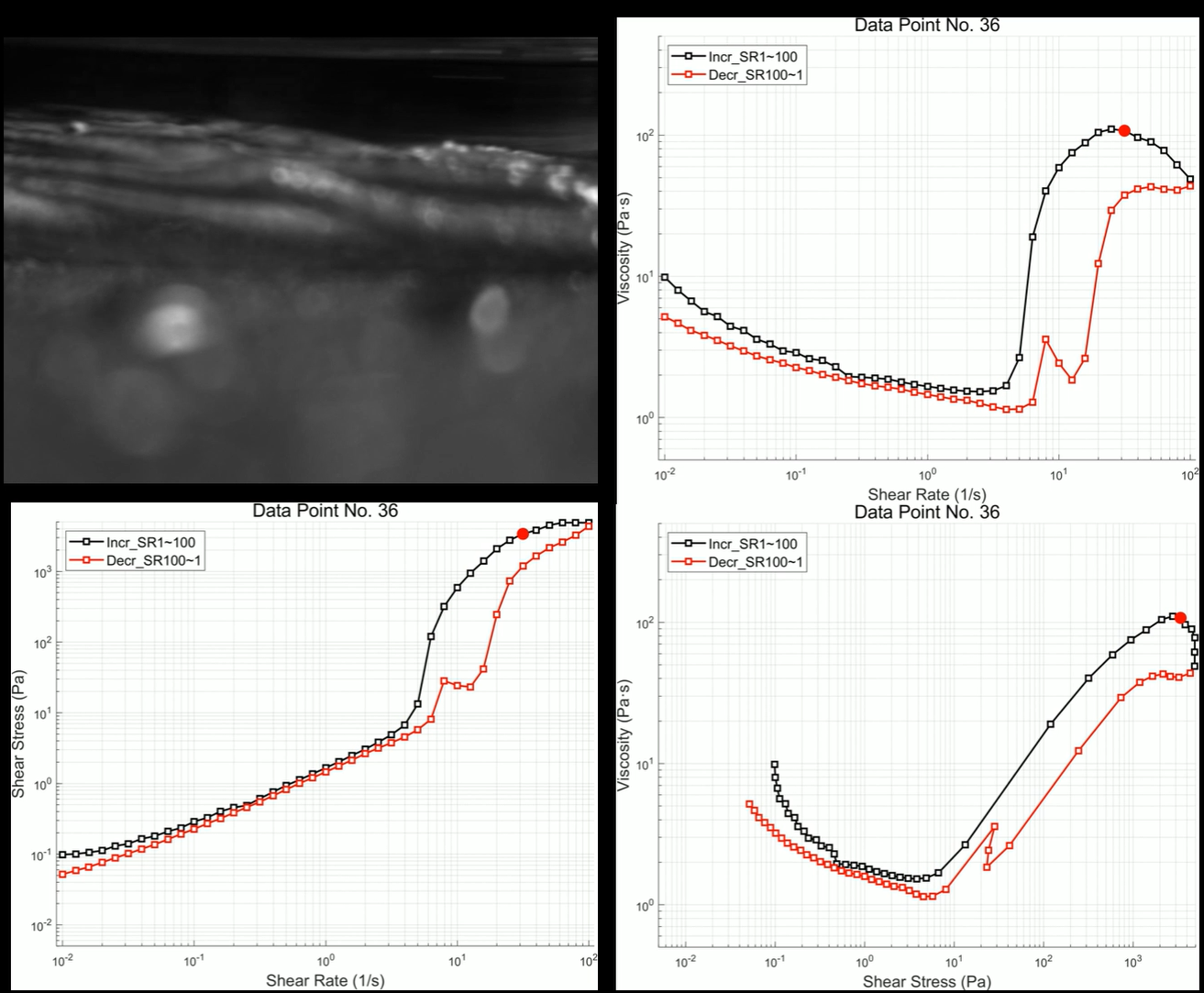


Video S2. Synchronized side-view observations aligned with rheological measurements. Gap distance 0.25 mm & rough surface at the bottom.


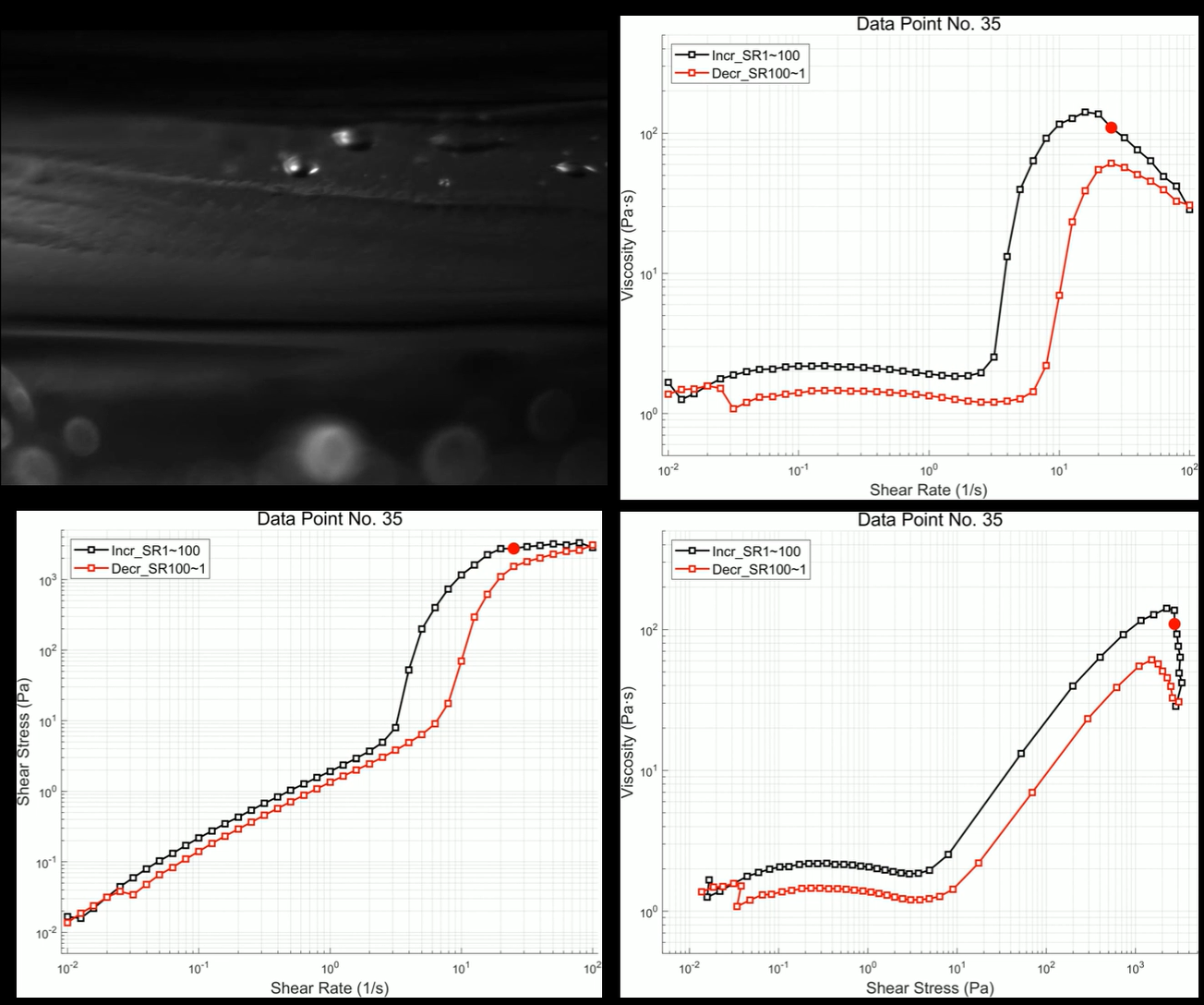


Video S3. Synchronized side-view observations aligned with rheological measurements. Gap distance 0.25 mm & smooth surface at the bottom.
